# Double-Transition-Metal Mo_2_Ti_2_C_3_ Mxene-Polyaniline Composites for High-Performance Supercapacitors

**DOI:** 10.1021/acsomega.6c06468

**Published:** 2026-09-10

**Authors:** Nisha H. Makani, Shrabani De, Mia Thompson, Peshal Karki, Bishnu Prasad Bastakoti, Bhoj Raj Gautam

**Affiliations:** † Department of Chemistry, Physics, and Materials Science, 3338 Fayetteville State University, Fayetteville, North Carolina 28301, United States; ‡ Department of Chemistry, 3616 North Carolina A&T State University, Greensboro, North Carolina 27411, United States

## Abstract

The optimized integration of two-dimensional (2D) MXene and conducting polymer composites offers an effective strategy for developing high-performance supercapacitor electrodes, owing to their exceptional flexibility, large surface area, and high capacitance. Herein, the double-transition-metal (DTM) MXene Mo_2_Ti_2_C_3_ (M), derived from its MAX phase Mo_2_Ti_2_AlC_3_ via selective chemical etching, was combined with polyaniline (PANI) through in situ polymerization to obtain composites with varying M/PANI ratios to enhance electrochemical performance. The structural, chemical, and morphological properties of the synthesized materials were systematically investigated using X-ray diffraction (XRD), Fourier transform infrared spectroscopy (FTIR), scanning electron microscopy (SEM), and X-ray photoelectron spectroscopy (XPS), confirming the successful formation of MXene and its effective integration with PANI fibers. The electrochemical behavior of all the prepared electrode materials was investigated using cyclic voltammetry (CV), galvanostatic charge–discharge (GCD), and electrochemical impedance spectroscopy (EIS) in an organic electrolyte. As a result, the optimized composite exhibits a high specific capacitance of 704 F g^–1^ at a current density of 2 A g^–1^. Furthermore, an asymmetric supercapacitor device assembled using the optimized composite as the positive electrode delivers a specific capacitance of 384.9 F g^–1^ at 1 A g^–1^, along with an energy density of 104.8 Wh kg^–1^, and a power density of 1400 W kg^–1^. These results highlight the significant potential of DTM M/PANI nanocomposites as advanced electrode materials for efficient energy storage applications.

## Graphical abstract

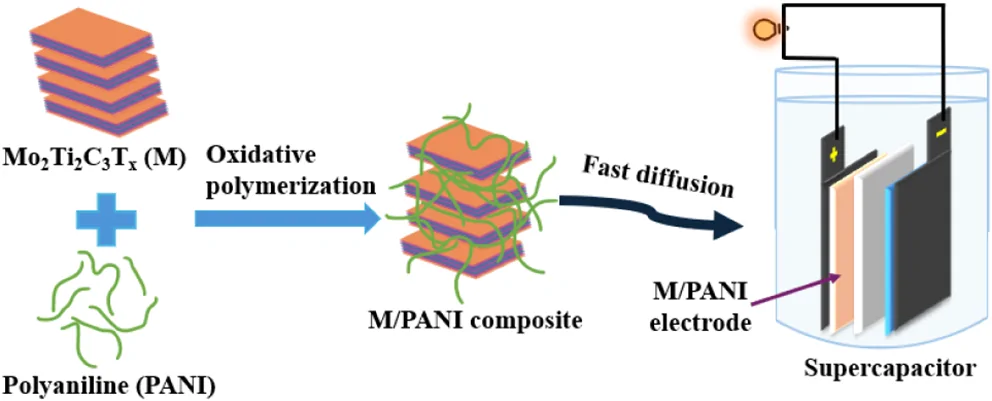

## 1 Introduction

According to the International Energy Agency (IEA), global electricity demand has increased significantly, driven by infrastructure development in emerging economies and transportation.^1^ This continued growth in energy consumption highlights the need for advanced energy storage systems capable of efficient power management. Specifically, the rapid production of portable electronics and electric vehicles has intensified the demand for high-performance electrochemical energy storage devices. Among the available technologies, supercapacitors have attracted considerable attention due to their rapid charge–discharge kinetics and long-term cycling stability, enabling them to assist as promising substitutes to conventional batteries in applications requiring high power delivery, short-term backup systems, and portable or wearable electronics.^2^ However, their relatively low energy density compared to batteries remains a key limitation. To overcome this challenge, there is a need to develop advanced electrode materials that can store more charge efficiently.^3^ Various materials, including carbon-based materials,^4^ metal oxides,^5^ conductive polymers,^6^ MXenes,^7^ and metal-organic frameworks (MOFs),^8^ have been widely investigated to enhance the performance and energy-storage capability of supercapacitors.^2^ Supercapacitors store energy via electric double-layer capacitance (EDLC) and/or pseudocapacitive mechanisms, depending on the electrode material used.^9^ Carbon-based electrodes offer excellent rate capability and long stability, but their capacitance is relatively low, whereas pseudocapacitive materials, such as transition-metal compounds and conducting polymers, exhibit higher specific capacitance through rapid surface redox reactions.^6,10^

Two-dimensional (2D) transition metal carbides and nitrides, known as MXenes, have gained attention as next-generation electrode materials for energy storage applications.^11^ MXenes are derived from layered MAX phases (M_n+1_AX_n_, where M represents an early transition metal, A is a group 13–16 element, and X is carbon and/or nitrogen) through selective etching of the A layer.^12^ MXenes demonstrate significant pseudocapacitive behavior arising from redox-active surface functional groups (−OH, −O, −F) combined with high electrical conductivity.^13,14^ Furthermore, their layered structure and large specific surface area allow operative EDLC, and intrinsic hydrophilicity helps good dispersion and strong interfacial interaction with conducting polymers, facilitating efficient ion transport.^2,15^ Owing to these unique properties, they have emerged as promising electrode materials for advanced electrochemical supercapacitors. Among available MXenes, the double transition metal (DTM) Mo_2_Ti_2_C_3_(M) MXene has attracted attention in many applications.^16–22^ Nevertheless, issues such as nanosheet restacking and limited long-term stability persist even in DTM MXenes, restricting electrolyte accessibility and electrochemical performance.^23,24^ To overcome these limitations, hybridization with conductive polymers has developed as an effective strategy. Conducting polymers such as polyaniline (PANI),^25^ polypyrrole,^10^ polyindole,^26^ polythiophene,^27^ and poly­(3,4-ethylenedioxythiophene):poly­(styrenesulfonate) PEDOT: PSS^28^ offer additional pseudocapacitive redox activity, allowing enhanced faradaic charge storage. Among these, PANI is particularly attractive due to its high specific capacitance, cost-effectiveness, and environmental stability.^25^

Several studies have demonstrated improved electrochemical performance in MXene/PANI composites.^29–33^ However, a systematic understanding of composition-dependent structural development and interfacial interaction, particularly in DTM MXene-based systems, remains limited. The influence of PANI integration on interlayer spacing of MXene, crystallinity, and composite morphology requires systematic investigation to establish clear structure-property relationships. In this work, we report the synthesis of DTM MXene via a strong etching method, followed by the fabrication of M/PANI nanocomposites through in situ polymerization with varying PANI concentrations in the MXene matrix. By systematically tuning the MXene to PANI ratio, we investigate the influence of PANI incorporation on the structural and morphological properties of the composites. The introduction of PANI nanofibers significantly enhanced the electrochemical performance of MXene, delivering a specific capacitance of 643.9 F g^–1^ at a current density of 2 A g^–1^. Moreover, the optimized M/PANI electrode was assembled into an asymmetric supercapacitor that achieved a specific capacitance of 384.9 F g^–1^ at 1 A g^–1^, along with a maximum energy density of 104.8 Wh kg^–1^ and a power density of 1400 W kg^–1^, demonstrating effective energy storage capability.

## 2 Materials and Experimental Methods

### 2.1 Materials

Mo_2_Ti_2_AlC_3_ MAX phase powder was purchased from Sigma-Aldrich. Hydrofluoric acid (HF, 48 wt %) and hydrochloric acid (HCl, 37 wt %) were also obtained from Sigma-Aldrich. Aniline monomer (≥99.5%) and ammonium persulfate (APS, ≥98%) were procured from Merck. Polyvinylidene fluoride (PVDF) and carbon black were purchased from Alfa Aesar. Tetraethylammonium tetrafluoroborate (TEABF_4_, ≥99%) was purchased from Sigma-Aldrich. *N*-Methyl-2-pyrrolidone (NMP, ≥99.5%) was obtained from Merck. Dimethyl sulfoxide (DMSO) was purchased from Fisher Scientific, USA. All materials were used as received without further purification.

### 2.2 Materials Synthesis

#### 2.2.1 Mo_2_Ti_2_C_3_ MXene

Multilayer DTM MXene was synthesized via selective chemical etching of the Mo_2_Ti_2_AlC_3_ MAX phase. In a Teflon container, 0.5 g of Mo_2_Ti_2_AlC_3_ powder was gradually added to 14 mL of hydrofluoric acid (HF, 48 wt %) under continuous magnetic stirring. The reaction mixture was maintained at 60 °C in an oil bath for 7 days to ensure complete removal of the Al layer. After etching, the resulting solution was repetitively washed with deionized (DI) water and centrifuged at 3500 rpm until the supernatant reached a pH of approximately 6. Following synthesis, the precipitated product was isolated and extensively washed with deionized water and ethanol to ensure the complete removal of unreacted precursors and impurities. The cleaned material was then dried in a vacuum oven at 60 °C for 24 h to obtain multilayer MXene powder.

#### 2.2.2 Polyaniline (PANI)

PANI was synthesized via oxidative polymerization of aniline in an acidic medium. Briefly, 1 mL of aniline monomer was dissolved in 50 mL of 0.1 M HCl under continuous stirring to form a homogeneous solution. Separately, 2.5 g of APS was dissolved in 50 mL of DI water to prepare the oxidant solution. The APS solution was then gradually added dropwise to the aniline solution under constant stirring while maintaining the reaction temperature between 0 °C and 3 °C using an ice bath. The polymerization was allowed to proceed under continuous stirring at a low temperature to ensure controlled formation of PANI.

#### 2.2.3 MXene/Polyaniline (M/PANI) Composites

M/PANI composites were prepared via an in-situ polymerization approach, and a schematic illustration is shown in Figure 1. In a typical procedure, 100 mg of MXene powder was dispersed in 50 mL of 0.1 M HCl and sonicated until a uniform suspension was obtained. The required amount of aniline solution was then added to the MXene dispersion, followed by dropwise addition of APS solution under continuous stirring at 0 °C–3 °C. The mass ratio (w/w) of M to PANI was systematically varied as 1:10, 1:5, and 1:2.5 to obtain M/PANI-10, M/PANI-5, and M/PANI-2.5 composites, respectively. For example, the 1:10 composite was prepared by adding 1 mL of aniline (density ≈ 1.02 g mL^–1^) together with the corresponding amount of APS (2.5 g) to initiate polymerization. The reaction mixtures were kept under refrigeration overnight to ensure complete polymerization and to control the polymerization rate. Following synthesis, the precipitated product was isolated and extensively washed with DI water and ethanol to remove unreacted precursors and impurities. The cleaned material was then dried at 60 °C to obtain the final M/PANI composite powders.

**Figure 1:**
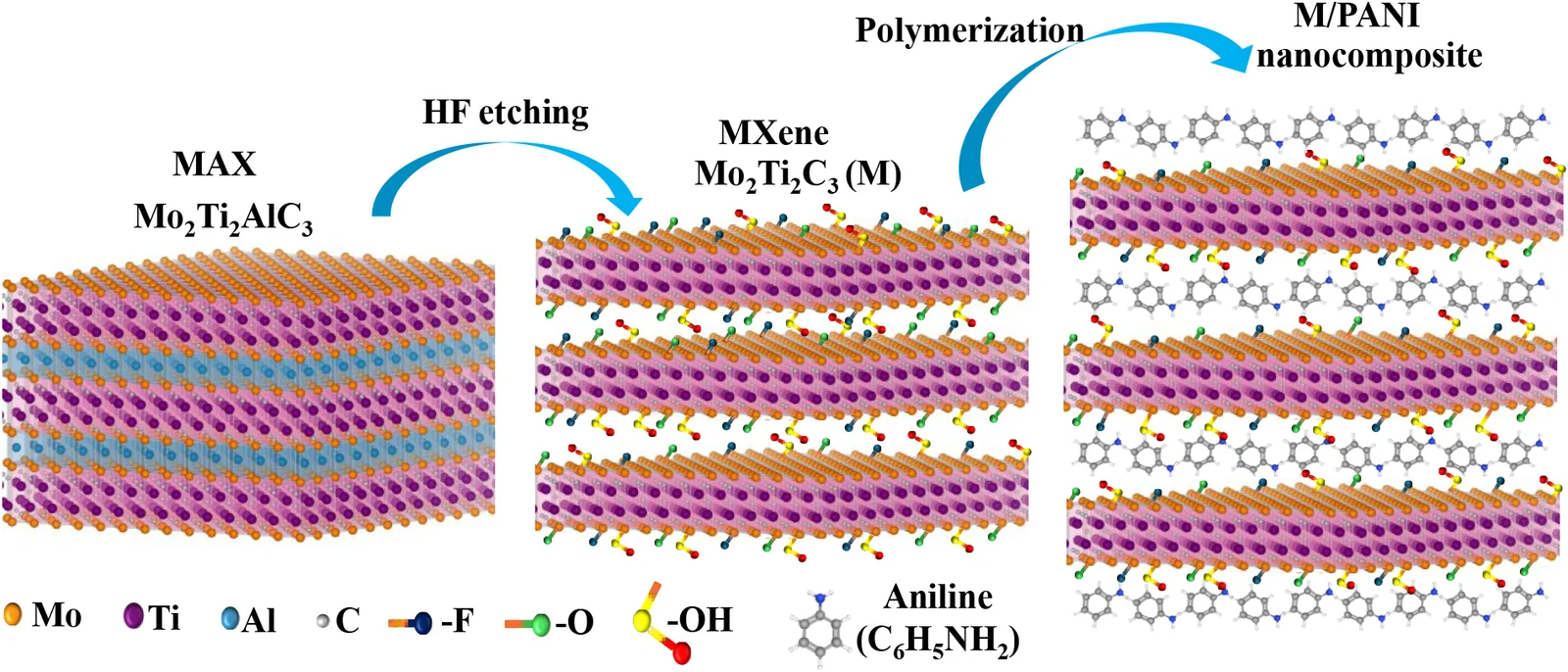
Schematic diagram of the material synthesis method for M/PANI composites. First, MXene was obtained by selective etching of the Al layers from the MAX phase with HF. Then, the M/PANI composite was synthesized via in situ chemical oxidative polymerization of aniline on MXene layers, using APS as the oxidizing agent.

## 3 Results and Discussion

### 3.1 Morphology and Structure

Figure 2a presents the XRD patterns of the Mo_2_Ti_2_AlC_3_ MAX phase and its MXene. The 2θ of the characteristic (002) peak shifts from 7.6° for the MAX phase to 5.4° for the MXene after etching, equivalent to an increase in interlayer spacing (*d*) along the *c* direction from 11.62 Å to 16.36 Å. This expansion in *d* spacing indicates successful etching of the Al layer and the incorporation of surface terminations such as −OH, −F, and −O.^34,35^ Furthermore, the Al-related peak at 41.5° (106) plane shows a significant reduction in intensity, indicating effective removal of the Al layer and the successful conversion of the MAX phase into MXene.^36,37^ The SEM image of the synthesized MXene sheets (Figure 2b) reveals sheet-like morphology. Elemental mappings as shown in Figure 2(c1–c5) further demonstrate the uniform distribution of Mo and Ti, along with F, O, and C signals, confirming successful MXene formation and surface functionalization. HF etching removes the A-layer from the MAX precursor and generates surface terminations such as −F, −OH, and −O. Although these functional groups enhance the hydrophilicity, the exposed transition-metal sites and intercalated water can promote gradual oxidation during storage. The XRD patterns shown in Figure S1 compare the freshly prepared MXene with the sample stored for 9 months. The principal diffraction features are largely preserved, and no additional sharp diffraction peaks corresponding to TiO_2_ or Mo crystalline oxide phases are observed, suggesting that no significant bulk oxidation occurred during storage. However, the reduced intensity of the characteristic (002) diffraction peak in the aged sample is primarily attributed to the partial restacking of the MXene nanosheets and the gradual loss of long-range stacking order during storage.

**Figure 2:**
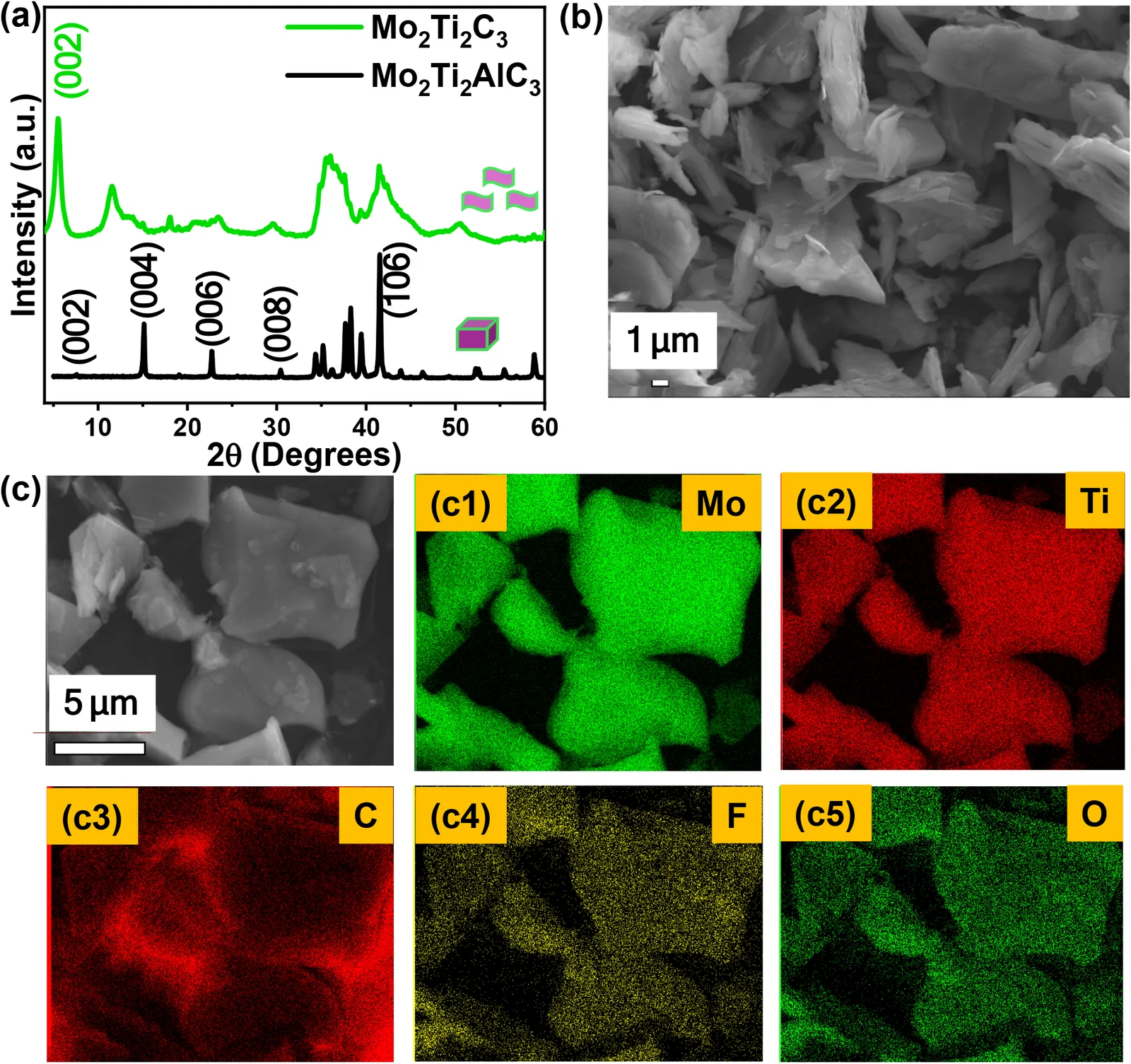
(a) XRD patterns of the Mo_2_Ti_2_AlC_3_ MAX phase and the Mo_2_Ti_2_C_3_ MXene, (b) SEM image of the synthesized MXene, (c) selected region for elemental mapping analysis, (c1–c5) elemental distribution maps of Mo, Ti, C, F, and O, respectively.

XRD patterns of pristine MXene, pure PANI, and their composites with changing MXene-PANI ratio are shown in Figure 3a. Pure PANI exhibits characteristic diffraction peaks at 2θ values of 8.9°, 15.2°, 20.2°, and 25.2°, corresponding to the (001), (011), (020), and (200) planes, respectively.^38^ The low-angle peak at ∼6.7° arises from interchain separation and reflects the long-range ordering of polymer chains.^39^ These peaks are also observed in the composite samples, confirming successful in-situ polymerization of PANI in the M/PANI system. The diffraction peaks of MXene are less pronounced in composites M/PANI-10 and M/PANI-5 and become more distinct in M/PANI-2.5. This is due to the increased PANI content, which can mask the MXene signal in M/PANI-10 and M/PANI-5, as well as to the effective coverage or wrapping of MXene by the PANI matrix. The apparent crystallite size and interlayer spacing of the pristine M and M/PANI composites were estimated from the (002) diffraction peak using the Scherrer equation and Bragg’s law, respectively. As summarized in Table S1, the pristine M exhibited an apparent crystallite size of 10.88 nm. Upon incorporation of PANI, the apparent crystallite size varied significantly with composition. The M/PANI-10 composite exhibited the largest crystallite size (28.78 nm). In contrast, the M/PANI-5 and M/PANI-2.5 composites showed reduced crystallite sizes of 4.36 and 7.98 nm, respectively. Notably, a shift in the (002) peak is clearly visible toward lower angles, from 5.4° to 4.65° in the M/PANI-5 composite. This shift corresponds to an increase in the interlayer spacing from 1.63 to 1.86 nm, indicating more effective intercalation of PANI chains between the MXene layers in the M/PANI-5 composite than in the other composites. However, at lower PANI loading (M/PANI-2.5), the interlayer spacing decreased to 1.51 nm.

**Figure 3:**
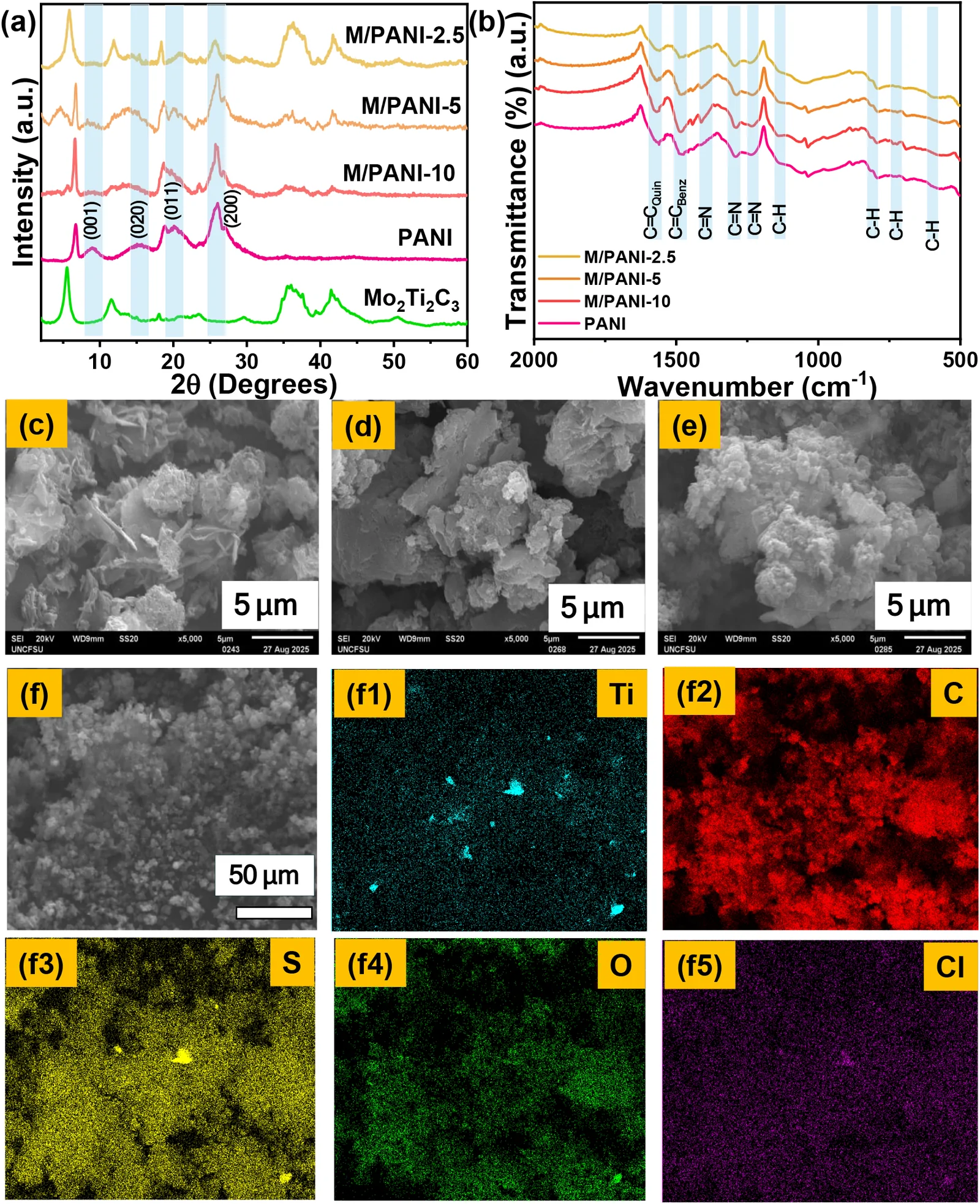
(a) XRD patterns and (b) FTIR spectra of all prepared samples, including pristine MXene, PANI, and their nanocomposites (M/PANI) with different ratios, SEM images of (c) M/PANI-10, (d) M/PANI-5, and (e) M/PANI-2.5; (f) SEM image of the selected region of the M/PANI-5 sample for elemental mapping, and (f1–f5) corresponding elemental maps of Ti, C, S, O, and Cl present in the nanocomposite.

Figure 3b shows the FTIR spectra of PANI and M/PANI nanocomposites. The spectra provide insight into the functional groups present and the interaction between MXene nanosheets and the PANI matrix. The FTIR spectrum of PANI shows distinct bands at 1568 and 1488 cm^–1^, attributed to the C=C stretching vibrations of the quinoid and benzenoid rings, respectively.^40^ Additional bands observed at 1384 and 1296 cm^–1^ are assigned to C–N stretching modes of aromatic ring, indicating the presence of C–N linkages within quinoid and benzenoid structures.^40^ A band at 1130 cm^–1^ is associated with aromatic C–H in-plane bending, while the peaks at 611, 719, and 804 cm^–1^correspond to aromatic C–H out-of-plane bending vibrations.^40^ These characteristic features are in good agreement with reported studies, confirming the formation of PANI. Importantly, these bands are retained in all composite spectra, demonstrating the successful incorporation of PANI into the MXene structure. The Raman spectra of the Mo_2_Ti_2_C_3_, polyaniline (PANI) and their composites are shown in Figure S2. The Raman spectrum of Mo_2_Ti_2_C_3_ exhibits four characteristic peaks. The broad peak near 258 cm^–1^ indicates vibrations of Mo, Ti and C atoms, as well as contributions from surface termination groups. The peaks located at approximately 400 cm^–1^, 605 cm^–1^, and 820 cm^–1^ are attributed to the out-of-plane vibrations of Mo and Ti atoms,^41,42^ in-plane vibration of the sub-surface carbon atoms^42,43^ and the Mo–O–Mo stretching vibrations^42,44^ respectively. Similarly, the Raman spectrum of PANI consists of two broad bands at approximately 1385 cm^–1^ and 1575 cm^–1^ corresponding to the νC–N stretching vibration of polaronic structures and the νC–N stretching mode associated with quinoid units, respectively.^45^ The Raman spectra of the M/PANI composites retain the characteristic bands of both Mo_2_Ti_2_C_3_ and PANI, confirming that the structural features of each component are preserved even after composite formation. As the PANI content increases from M/PANI-2.5 to M/PANI-10, the intensity of the Mo_2_Ti_2_C_3_ Raman bands gradually decreases, while the characteristic PANI bands become progressively more pronounced. This systematic variation in the relative peak intensities reflects the changing composition of the composites and provides further evidence for the successful incorporation of PANI into the Mo_2_Ti_2_C_3_ MXene matrix.

The surface morphology of the synthesized M/PANI and PANI was examined using SEM. The SEM images of PANI (Figure S3a, b) with lower and higher magnification reveal a uniform fibrous interconnected network structure, which is characteristic of chemically oxidized PANI synthesized under low-temperature conditions.^46^ The formation of well-defined nanofibers, along with EDS analysis (Figure S3c–d) showing the presence of C and N, confirms the successful polymerization of aniline into PANI and indicates controlled polymer growth during the process. Figure 3(c–e) presents SEM analysis of all three M/PANI compositions (10, 5, and 2.5), respectively, which reveals a clear evolution in morphology with increasing MXene concentration into the PANI matrix. SEM image of M/PANI-10 shows distinguished sheets (Figure 3c, Figure S4a, b). In M/PANI-5, the morphology transitions into a mixture of exposed sheet-like structures and localized agglomerates (Figure 3d, (Figure S3a, b). Further decreasing the PANI content in M/PANI-2.5 results in pronounced agglomeration due to insufficient polymer acting as a spacer, promoting restacking of sheets (Figure 3e, Figure S4a, b). Elemental mapping and EDS spectra of M/PANI-10 (Figure S4d), M/PANI-5 (Figure 3f, Figure S5d), and M/PANI-2.5 (Figure S6d) nanocomposites confirm the presence of Mo, Ti, C, O, Cl, and S in the nanocomposites. The sulfur originates from sulfate ions introduced during the oxidative polymerization of aniline, which act as dopant anions stabilizing the protonated PANI backbone. The detected chlorine is attributed to residual chloride species from the M/PANI synthesis process. The weak or absent Mo signal in the M/PANI-10 and M/PANI-5 composite can be attributed to the encapsulation of MXene nanosheets by the PANI matrix, as well as the overlap of Mo and S energy peaks.

The HRTEM image of the M/PANI-5 nanocomposite clearly reveals PANI nanofibers wrapped with 2D MXene layers (Figure 4a), which can be attributed to the growth of PANI over the MXene matrix. Figure 4b shows a selected area electron diffraction (SAED) pattern exhibiting diffuse ring features, attributed to the polycrystalline MXene phase, however, their intensity is significantly reduced due to the dominant amorphous PANI matrix, which masks the crystalline features. Furthermore, elemental mapping analysis confirms the homogeneous distribution of all constituent elements, including Mo, Ti, C, N, O, and Cl, throughout the sample (Figure 4c­(c1–c6)). The PANI nanofibers wrapped with 2D MXene layers, along with uniform elemental dispersion, demonstrate the successful formation of the M/PANI nanocomposite with strong interfacial interaction.

**Figure 4:**
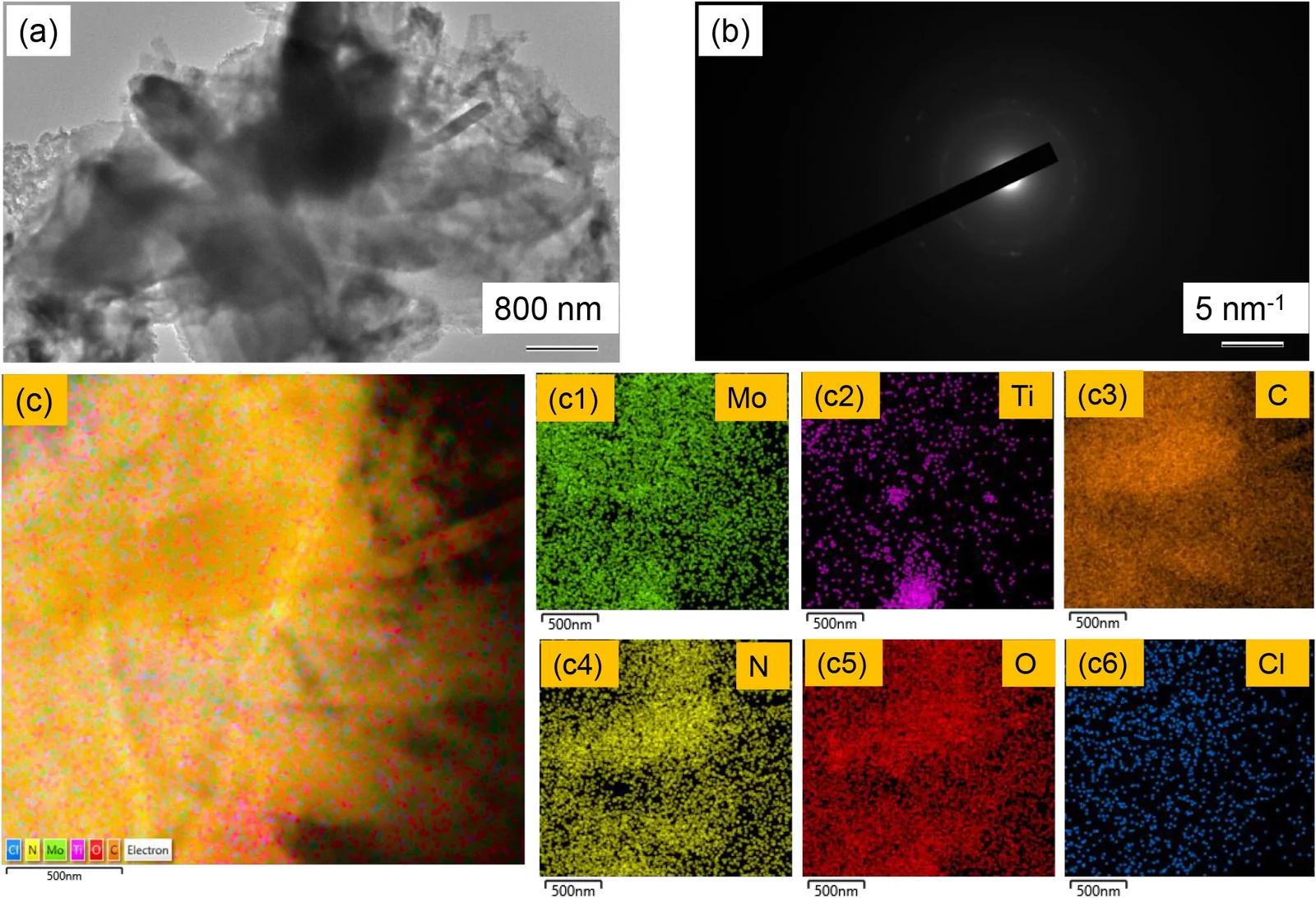
(a) HRTEM image, (b) SAED pattern, and (c) EDS elemental layered mapping image and (c1–c6) mapping of elements (Mo, Ti, C, N, O, and Cl) of the M/PANI-5 nanocomposite.

X-ray photoelectron spectroscopy (XPS) was used to examine the surface chemical composition and bonding states of the synthesized MXene (Figure S7) and the M/PANI-5 nanocomposite (Figure 5). The survey spectrum of pristine MXene confirms the presence of Mo, Ti, C, O, and F elements (Figure S7a). For the M/PANI-5 composite, the survey spectrum confirms the presence of Mo, Ti, C, O, S, and N elements (Figure 5a). The surface carbon concentration increased from 49.2 to 79.1 at. %, while the Ti, Mo and F concentrations decreased from 5.9, 9.7, and 16.6 at. % to 0.7, 2.1, and 0.4 at. %, respectively. These changes are consistent with attenuation of the MXene-derived signals by coverage with a carbon-rich polymer phase. However, accurate quantification of nitrogen was not possible because the N 1s region overlaps with the Mo 3p_3/2_ signal.

**Figure 5:**
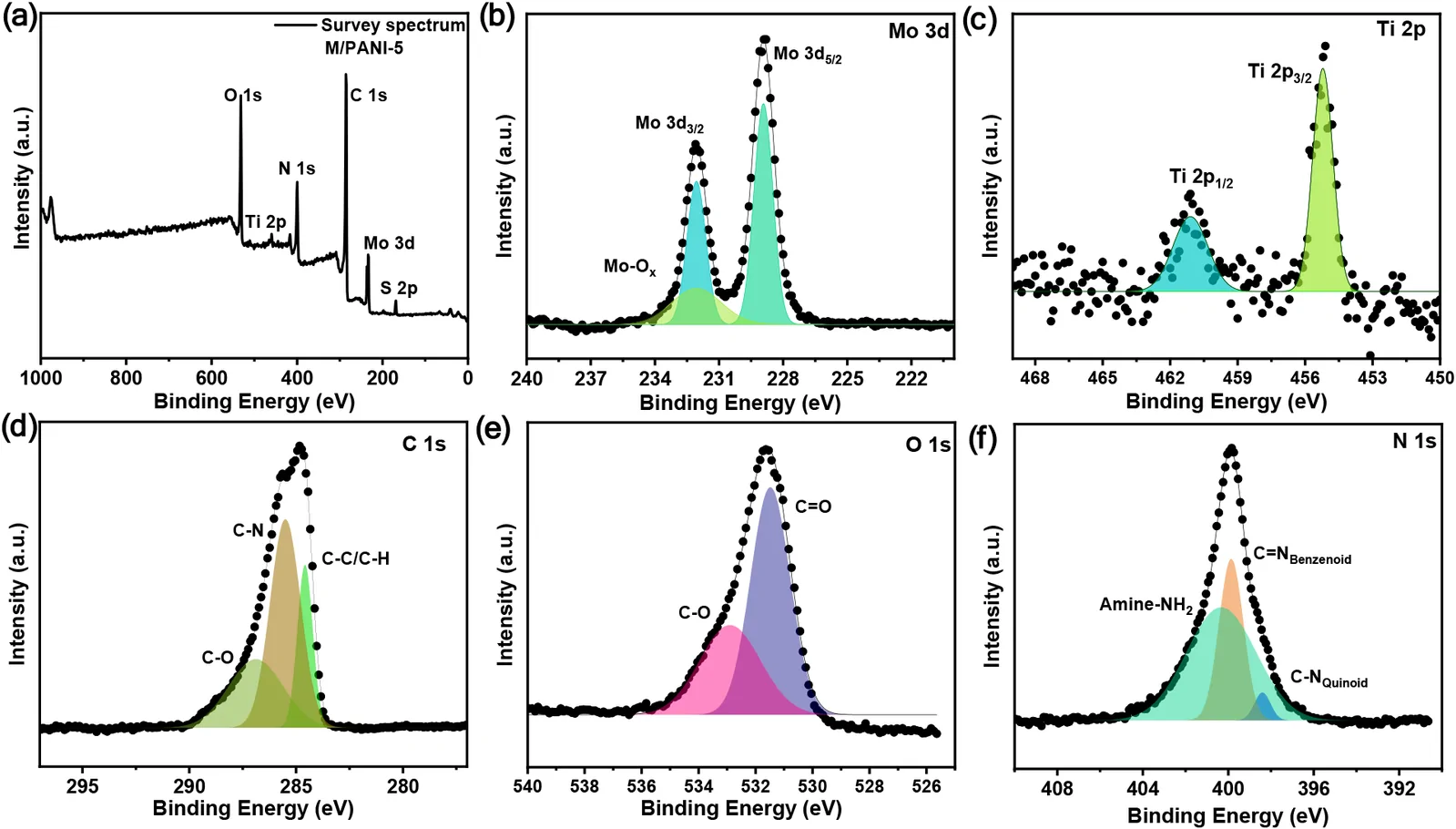
(a) XPS survey spectrum of M/PANI-5 composite, and the corresponding high-resolution spectra of (b) Mo, (c) Ti, (d) C, (e) O, and (f) N, respectively.

In the high-resolution Mo 3d spectrum (Figure S7b), the peaks located around 229.04 eV, and 230.2 eV correspond to the Mo–C bonds (Mo 3d_5/2_ and Mo 3d_3/2_), confirming the carbide structure of the MXene.^22^ Additional peaks observed in the range of (232.27–235.6) eV are attributed to oxidized Mo species, which typically form on the MXene surface during the etching process and subsequent exposure to air.^22,41,47^ The Mo 3d spectrum of the M/PANI-5 composite exhibit characteristic Mo 3d_5/2_ and Mo 3d_3/2_ peaks along with Mo–O_*x*_ components at 228.92, 232.10, and 233.25 eV, respectively (Figure 5b). The Ti 2p spectrum (Figure S7c) exhibits components near 455.01 eV associated with Ti–C bonds, along with peaks around 458.53 eV corresponding to Ti–O species, indicating partial surface oxidation.^38,47,48^ The Ti 2p spectrum also displays characteristic Ti 2p_3/2_ and Ti 2p_1/2_ peaks at 455.21 and 461.10 eV, respectively (Figure 5c). The C 1s spectrum (Figure S7d) shows characteristic peaks for carbide carbon (C–Ti/C–Mo) at a lower binding energy (282.37 eV). The peak observed at 284.5 eV corresponds to sp^2^ carbon C–C/C=C contributions.^48^ The C 1s spectrum (Figure 5d) of the M/PANI-5 nanocomposite (Figure 5d) shows noticeable changes compared to MXene and can be deconvoluted into three chemically distinct components. The prominent peaks corresponding to C–C/C–H (284.5 eV), C–N (285.5 eV), and C–O (286.8 eV) confirm the formation of PANI through the polymerization of aniline.^38^ In addition, the presence of a peak near ∼686 eV in the F 1s region (Figure S7e) confirms the formation of metal-F surface terminations resulting from HF etching,^49^ while the O 1s for MXene and M/PANI-5 (Figures S7f, 5e) peaks near ∼530–531 eV correspond to metal–oxygen bonds and hydroxyl terminations on the MXene surface.^50^ In addition to the elements observed in pristine MXene, the M/PANI-5 composite spectrum exhibits the appearance of an N 1s (Figure 5a) peak can be deconvoluted into three distinct peaks at 400.3, 399.8, and 398.4 eV, equivalent to positively charged nitrogen species, benzenoid amine (−N–H), and quinoid imine (−N=) groups, respectively. These features confirm the presence of polyaniline on the surface of MXene.^38,51^

### 3.2 Electrochemical Performance

#### 3.2.1 Supercapacitor Application

First, a three-electrode setup in a 1 M TEABF_4_/DMSO electrolyte was used to assess the electrochemical performance of the produced samples within a potential window of 0.0–1.1 V. Figure 6a compares the CV curves of MXene, PANI, M/PANI-10, M/PANI-5, and M/PANI-2.5 at 20 mV s^–1^. The quasi-rectangular CV profile of pristine Mo_2_Ti_2_C_3_, which lacks identifiable redox peaks, indicates dominant electric double-layer capacitance (EDLC) resulting from reversible adsorption/desorption of TEA^+^ ions at the MXene surface and interlayer regions. The hydrophilic surface terminations (−O, −OH, −F) of MXene, which promote quick ion transport but contribute little faradaic activity, allow this capacitive behavior. PANI, on the other hand, exhibits distinct redox peaks at roughly 0.69 V (anodic) and 0.42 V (cathodic), which represent the reversible transition between the conjugated polymer chains’ polaronic and polycationic states.^52^ These redox reactions contribute to pseudocapacitive charge storage by oxidizing and reducing PANI’s nitrogen-containing functional groups.^52^ After composite formation with Mo_2_Ti_2_C_3_, the redox features become more pronounced in M/PANI-10 and M/PANI-5, followed by minor potential shifts and further peak broadening, indicating a significant interfacial interaction between MXene sheets and polymer chains. This interaction enhances electron transport and modulates polymer redox kinetics, enabling simultaneous EDLC at MXene interfaces and faradaic reactions within the polymer matrix. The decreased redox peaks for M/PANI-2.5 indicate a lower PANI content, leading to a charge-storage mechanism mostly controlled by MXene-based EDLC. M/PANI-5 shows the best balance between capacitive and pseudocapacitive contributions across all compositions, as evidenced by its greatest CV area and highest current responsiveness. The intercalation of PANI between MXene layers suppresses nanosheet restacking and establishes continuous conductive pathways, thereby facilitating rapid ion diffusion and charge-transfer kinetics. The GCD curves recorded at 2 A g^–1^ (Figure 6b) further confirm these observations. M/PANI-5 shows the maximum specific capacitance of 704 F g^–1^, surpassing Mo_2_Ti_2_C_3_ (264 F g^–1^), PANI (427.6 F g^–1^), M/PANI-10 (408 F g^–1^), and M/PANI-2.5 (484.5 F g^–1^). To ensure reproducibility, the electrochemical analysis were performed three times, and the reported specific capacitances have an error range of ±20 F g^–1^. The outstanding efficiency of M/PANI-5 arises from the synergistic integration of MXene-derived EDLC and uniform PANI coating with robust interfacial contacts that optimize electrochemically active sites and improve charge-storage efficiency which enables reversible polymer redox reactions.

**Figure 6:**
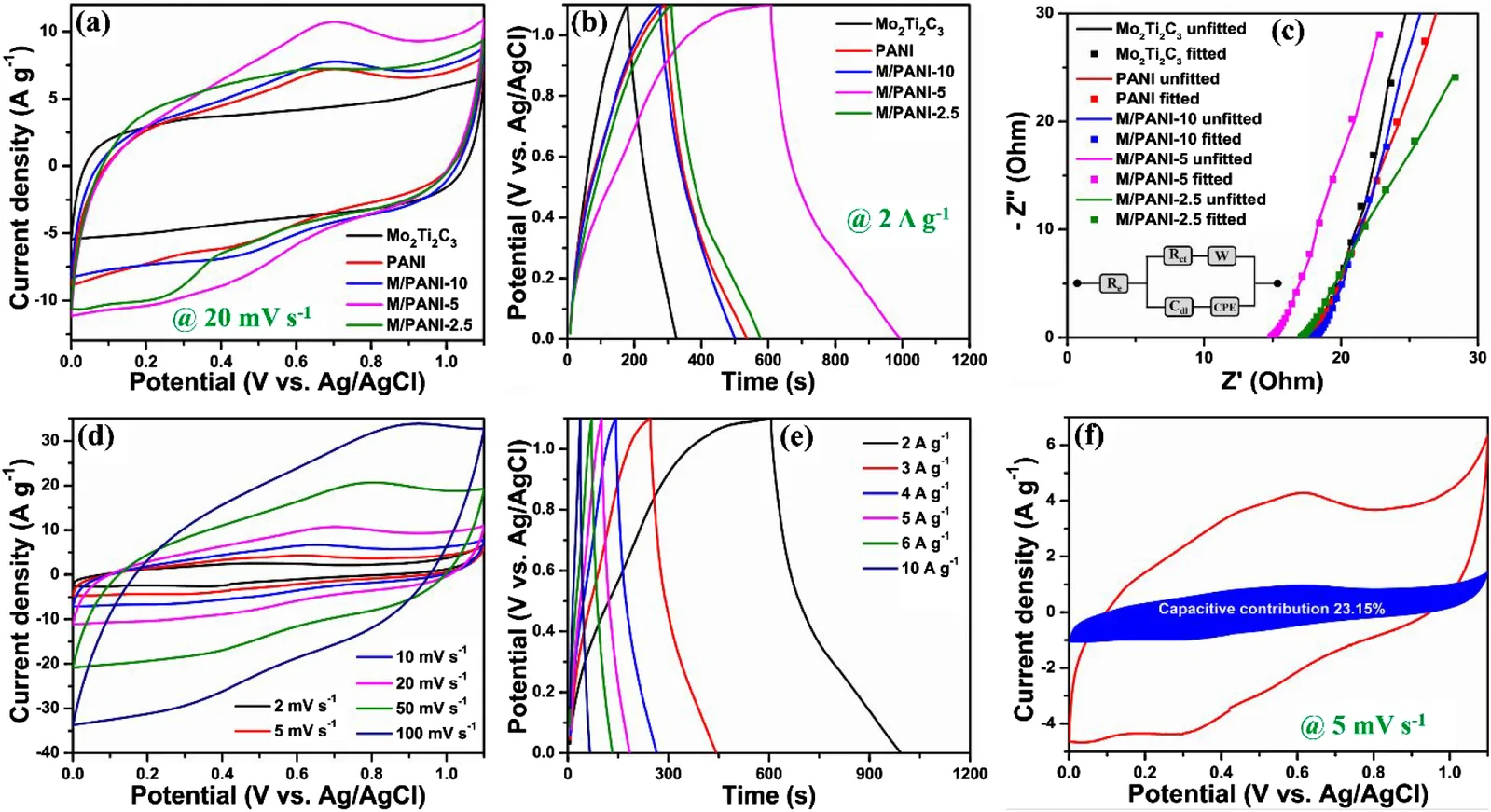
Comparison of (a) CV at 20 mV s^–1^, (b) GCD at 2 A g^–1^, (c) Nyquist plot, inset of (c) depicts the fitting circuit; (d) CV at various scan rates, (e) GCD at various current densities, and (f) capacitive output in CV plot at 5 mV s^–1^ of M/PANI-5.

The charge-transfer kinetics and interfacial capacitive properties of the synthesized electrodes were examined using EIS. The Nyquist graphs in Figure 6c display the imaginary impedance (−Z′) vs real impedance (Z′) at open-circuit potential over the frequency range of 1–10^6^ Hz. The equivalent series resistance (R_e_) in the high-frequency domain is represented by the intercept on the Z′ axis and consists of the active material’s intrinsic resistance, electrolyte resistance, and contact resistance at the electrode-current collector interface. The charge-transfer resistance (R_ct_), which reflects the kinetics of electron transport and ion exchange at the electrode–electrolyte interface, is represented by the depressed semicircle seen in the high–mid frequency range.^26^ Better electrical conductivity and more effective interfacial charge transfer are indicated by a smaller semicircle diameter. The double-layer capacitance (C_dl_), which is connected to efficient ion transport and charge accumulation at the electrode surface, is represented by the nearly vertical line that approaches the imaginary axis in the low-frequency region and denotes ideal capacitive behavior. Figure 6c shows the experimental and fitted Nyquist charts as well as the equivalent circuit model, and Table 1 summarizes the extracted parameters. The standard error for each fitting lies within the range of e^–3^. The M/PANI-5 composite has the lowest R_e_ and R_ct_ values of any sample, indicating faster interfacial charge-transfer kinetics and decreased internal resistance. The consistent PANI coating on Mo_2_Ti_2_C_3_ is responsible for this improvement, which enhances the electrical interaction between MXene sheets and the electrolyte by creating continuous conductive pathways. Additionally, M/PANI-5 displays the highest C_dl_ value, evidenced by the steeper low-frequency slope, indicating a larger electrochemically active surface area and improved ion accessibility. The combined reduction in R_e_ and R_ct_, along with the increase in Cdl, confirms that M/PANI-5 provides better capacitive performance, faster electron transfer, and improved ion transport, making it the most efficient electrode among the compositions investigated.

**Table 1:** Parameters Obtained by Fitting Nyquist Plots of the Synthesized Samples

Samples	R_e_ (Ω)	R_ct_ (Ω)	C_dl_ (F)	W (Ω·s^–1/2^)	CPE (F·s^(a–1)^)
Mo_2_Ti_2_C_3_	17.6	2.7	0.048	145.5	0.163
PANI	17.59	2.27	0.039	99.21	0.113
M/PANI-10	17.84	1.64	0.037	66.8	0.195
M/PANI-5	14.86	0.195	0.052	78.31	0.172
M/PANI-2.5	17.06	0.772	0.040	31.2	0.134

To further assess the electrochemical behavior of the optimized M/PANI-5 electrode, CV and GCD study were conducted at changing scan rates and current densities. As shown in Figure 6d, the CV curves retain well-defined redox peaks across all scan rates, with the enclosed area increasing progressively at higher scan rates, indicating rapid ion transport and efficient charge-transfer kinetics. The preserved peak shapes confirm stable pseudocapacitive behavior arising from reversible PANI redox reactions in combination with TEA^+^ adsorption on the surface of MXene. The resultant GCD profiles (Figure 6e) show approximately symmetric charge–discharge curves with strong reversibility and great Coulombic efficiency. Longer discharge times at low current densities result from sufficient diffusion and complete utilization of redox-active sites, whereas shorter discharge times at higher current densities are attributed to diffusion limitations under fast charge–discharge conditions. Crucially, even at 6 A g^–1^, M/PANI-5 maintains a specific capacitance of 336 F g^–1^, demonstrating its exceptional rate capability and appropriateness for high-power energy storage applications. The stability over long-term cycling of M/PANI-5 as shown in Figure S8c shows 84.5% retention of its prior specific capacitance after 10,000 charge–discharge cycles at 20 A g^–1^ depicting good reversibility during extended operation.

To further understand the charge-storage kinetics of the optimized M/PANI-5 electrode, a power-law analysis was applied using the relationship:^53^

1 $$i\left(V\right)=av^{b}$$

Here, a and b are modifiable constants, v is the scan rate, and *i* is the current response. The primary charge-storage mechanism can be inferred from the b-value: a diffusion-controlled process is indicated by b ≈ 0.5, whereas a surface-controlled capacitive behavior is indicated by b ≈ 1.0. From the log­(*i*) versus log­(v) plots (Figure S8), the anodic and cathodic peaks have computed b-values of 0.65 and 0.60, respectively. These intermediate values suggest that the M/PANI-5 electrode exhibits a hybrid charge-storage mechanism, involving capacitive-controlled contributions from the MXene framework and diffusion-controlled pseudocapacitive reactions associated with the PANI component.

The Dunn approach was used to quantitatively separate the diffusion-controlled and capacitive contributions using the relationship:^54^

2 $$i\left(V\right)=k_{1}v+k_{2}v^{1/2}$$

where *i­(V)* is the current response at a given potential,^32^ *v* is the scan rate, and *k*_*1*_ and *k*_*2*_ are potential-dependent constants corresponding to capacitive (*k*_1_v) and diffusion-limited (*k*_2_v^1/2^) processes, respectively. By plotting i/v^1/2^ against v^1/2^, different scan rates can be used to derive the respective contributions of surface-controlled and diffusion-controlled charge storage. As shown in Figure 6f, the capacitive output of M/PANI-5 at 5 mV s^–1^ is 23.15%, demonstrating that the total charge storage is dominated by diffusion-controlled processes. Specifically, diffusion-controlled contributions account for 84%, 76.85%, 70.13%, and 62.4% at scan rates of 2, 5, 10, and 20 mV s^–1^, accordingly. A high percentage of the diffusion-controlled behavior (>50%) indicates that electrochemical kinetics are mostly governed by bulk faradaic processes within the composite, while surface redox reactions and electric double-layer charging provide complementary capacitive support, particularly at higher scan rates.

To assess the practical performance of the optimized M/PANI-5 electrode, M/PANI-5 was used as the positive electrode and carbon black as the negative electrode to create an asymmetric supercapacitor (Figure 7a). For validation, the electrochemical efficiency of M/PANI-5 in positive potential range (0.0 to 1.1 V) and carbon black in negative potential window (0 to −0.8 V) are contrasted Figure S8c at 50 mV s^–1^ in three-electrode analysis. The operating voltage window was optimized by recording CV curves at 50 mV s^–1^ (Figure 7b) and GCD profiles at 1 A g^–1^ (Figure 7c) while gradually expanding the potential range. Based on curve stability and symmetry, a working voltage window of 0–1.4 V was established. The CV curves at various scan rates (Figure 7d) exhibit a quasi-rectangular shape with distinct redox peaks at approximately 0.6 V (anodic) and 0.3 V (cathodic), corresponding to reversible PANI redox reactions, confirming the coexistence of EDLC and pseudocapacitive behavior. The GCD profiles (Figure 7e) show quasi-symmetric curves with slight voltage plateaus, consistent with the mixed charge-storage mechanism. Using eqs S2–S4, the device exhibits a specific capacitance of 384.9 F g^–1^, an energy density of 104.8 Wh kg^–1^, and a power density of 1400 W kg^–1^ at 1 A g^–1^, demonstrating its promising potential for practical energy storage applications. The Ragone plot in Figure 7f demonstrates that the fabricated device (+//−) shows a significantly higher energy density and power density compared to previously reported supercapacitor systems including Ti_3_C_2_T_*x*_/PANI//Ti_3_C_2_T_*x*_/PANI (31.18 Wh kg^–1^ and 1079.3 W kg^–1^),^32^ Ti_3_C_2_/PANI nanotubes//Ti_3_C_2_/PANI nanotubes (25.6 Wh kg^–1^ and 153.2 W kg^–1^),^31^ Ti_3_C_2_T_*x*_/PANI/α-Fe_2_O_3_/MnO_2_/Ti_3_C_2_T_*x*_/PANI//Ti_3_C_2_T_*x*_/PANI/α-Fe_2_O_3_/MnO_2_/Ti_3_C_2_T_*x*_/PANI (17.45 Wh kg^–1^ and 1056.87 W kg^–1^),^30^ carbon cloth/Ti_3_C_2_T_*x*_/PANI/CoNi-LDH//carbon cloth/activated carbon (39.33 Wh kg^–1^ and 399.95 W kg^–1^),^29^ MnO_2_//Ti_3_C_2_T_*x*_/polypyrrole (35.3 Wh kg^–1^ and 486.1 W kg^–1^),^55^ polypyrrole/Ti_3_C_2_//polypyrrole/Ti_3_C_2_ (21.61 Wh kg^–1^ and 499.94 W kg^–1^),^56^ reduced graphene oxide/Ti_3_C_2_T_*x*_/polypyrrole//reduced graphene oxide/Ti_3_C_2_T_*x*_/polypyrrole (11.3 Wh kg^–1^ and 500 W kg^–1^),^57^ highlighting its improved energy-power efficiency.

**Figure 7:**
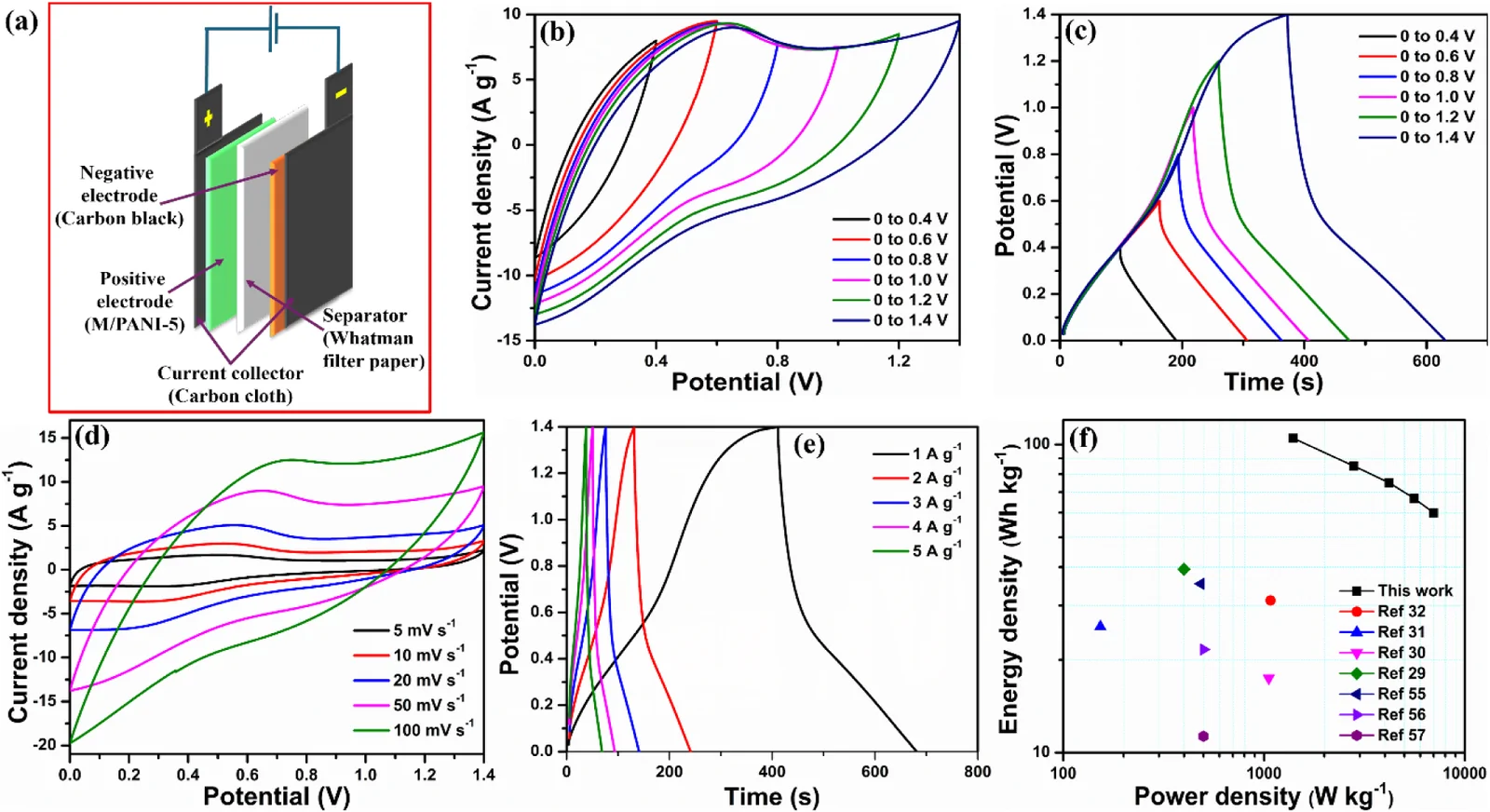
(a) Diagrammatic depiction of the assembled asymmetric supercapacitor, (b) CV profiles with an increasing voltage window at 50 mV s^–1^, (c) GCD profiles with increasing voltage range at 1 A g^–1^ of the fabricated supercapacitor, (d) CV at various scan speeds, (e) GCD at various current densities, and (f) Ragone plot contrasting the device performance with previous literatures.

The asymmetric supercapacitor’s Coulombic efficiency was measured at different current densities to determine charge-storage reversibility (Figure S8d). The Coulombic efficiency increased to around 95% at 5 A g^–1^ from 66% at 1 A g^–1^. The lower Coulombic efficiency at low current density stems from extended cycle durations that exacerbate parasitic side reactions.^58^ Charge recovery is greatly enhanced at higher current densities because these parasitic reactions are kinetically avoided by shorter cycle periods. The superior reversibility of the charge-storage mechanism is designated by the high Coulombic efficiency (>90%) attained above 3 A g^–1^, indicating highly accessible active sites in M/PANI-5 and stability of the supercapacitor. For a supercapacitor to be used practically, its long-term cycle stability is essential. As shown in Figure 8a, the assembled asymmetric device retains 85% of its initial specific capacitance after 10,000 charge–discharge cycles at 20 A g^–1^, with a stable Coulombic efficiency of ∼95%, indicating good reversibility and minimal parasitic reactions during extended operation. The steady capacitance decline is caused by the progressive loss of electrochemically active sites rather than irreversible charge-transfer events, and this trend suggests that the electrochemical reactions remain highly reversible throughout each cycle. Comparison of the CV curves at 100 mV s^–1^ before and after cyclic stability study (Figure 8b) reveals only a slight decrease in enclosed area and minor redox peak shifts, in line with the capacitance degradation that has been seen. This limited deterioration could result from progressive loss of active sites during repeated ion insertion-extraction procedures, partial pore blockage, or modest surface remodeling. Only slight alterations in the asymmetric supercapacitor’s electrochemical properties are visible in the impedance spectra taken prior to and following the cycling test (Figure 8c). The electrolyte and current collector maintain electrochemical stability throughout extended cycling, as evidenced by the almost constant solution resistance. Together with minor surface oxidation of the MXene nanosheets and a partial decrease in electrolyte accessibility, a gradual interfacial degradation resulting from repeated PANI chain expansion and shrinkage during redox cycling is responsible for a slight increase in the impedance at intermediate and low frequencies. By slightly raising the ion transport resistance and reducing the quantity of electrochemically active sites, these actions cause a slight fading of capacitance. Nonetheless, the overall impedance response does not change before or after cycling, demonstrating the continued high reversibility of the electrochemical reactions, and that the Mo_2_Ti_2_C_3_/PANI composite’s conductive network is successfully preserved.

**Figure 8:**
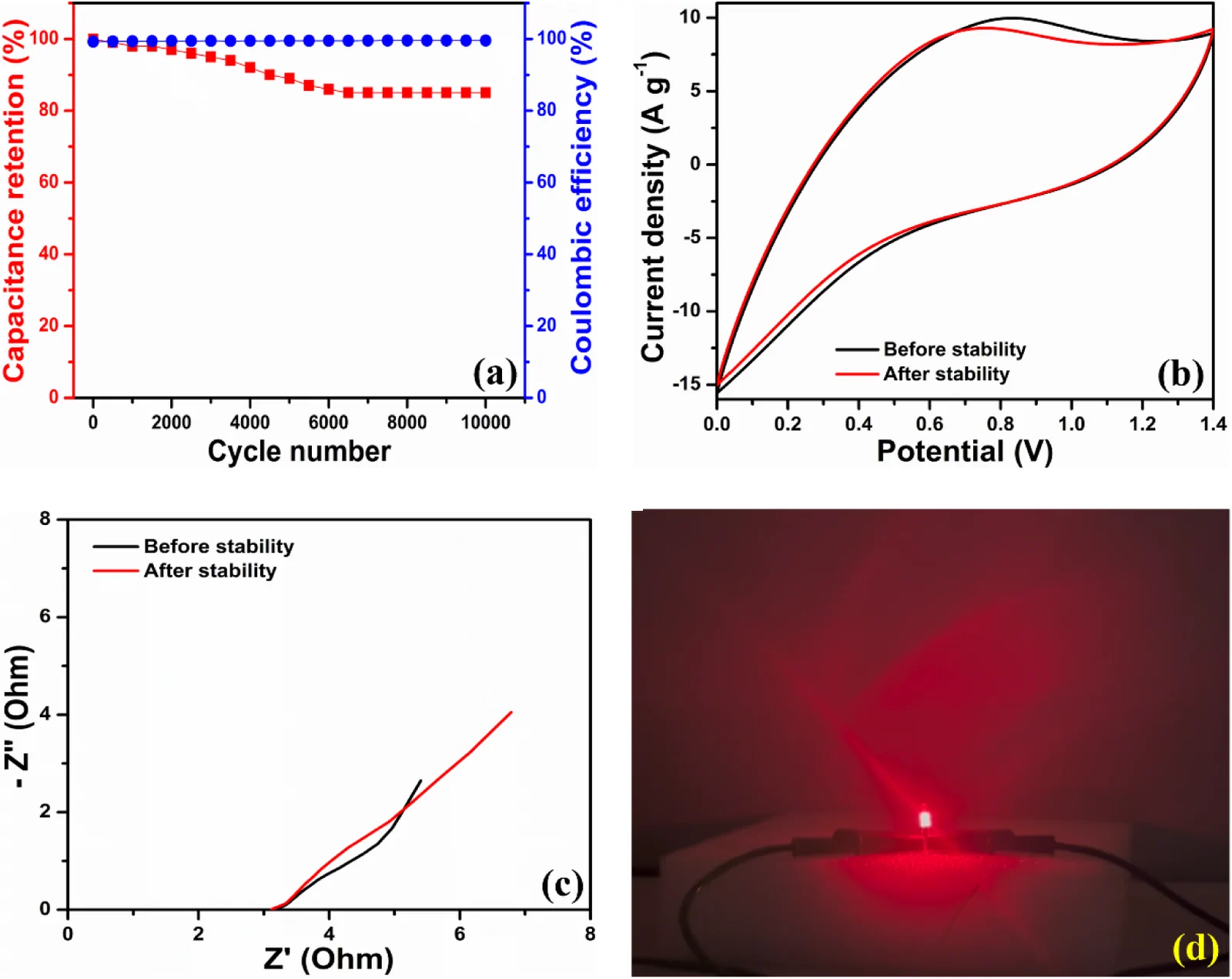
(a) Capacitance retention and Coulombic efficiency with number of cycles, (b) CV profile at 100 mV s^–1^ before and after stability analysis, (c) Nyquist plots before and after cyclic stability, and (d) illuminating a red LED with the prepared asymmetric supercapacitors.

Surprisingly, the energy density of this manufactured asymmetric device is relatively high compared with previously reported MXene-based supercapacitor devices (Table 2), demonstrating its superiority for real-time applications.

**Table 2:** Comparison of Performance of Our Asymmetric Supercapacitor with Recently Published Mxene-Based Supercapacitor Devices^a^

Supercapacitor devices (+//−)	Electrolyte	Voltage window (V)	Current density	C (F g^–1^)	E (Wh kg^–1^)	P (W kg^–1^)	% of C retention	No. of cycles	Refs
Ce-MOF/Mo_2_Ti_2_C_3_//rGO	6 M KOH	1.5	0.6 A g^–1^	164.5	51.43	1800	73.07	5000	^59^
MXene/ZnO//MXene/ZnO	1 M H_2_SO_4_	1.2	1 A g^–1^	32	6.33	600	97.2	12000	^60^
Fe_2_O_3_/MXene//AC	3M H_2_SO_4_ + 30 mM CuSO_4_	1.4	2 mV s^–1^	502.2	20.1	1292.5	-	-	^61^
V-PMOF@PANI// V-PMOF@PANI	1 M H_2_SO_4_	0.9	0.5 A g^–1^	118	30.30	450.85	86	10000	^62^
CoGa-LDH/MXene//AC	7 M KOH	1.6	0.25 A g^–1^	364.40	91.73	375	81.24	6000	^63^
CeO_2_–La_2_O_3_-MXene//AC	PVA/KOH	1.2	5 A g^–1^	156	31.33	3000	77	25000	^64^
AC//MnO_3_/MXene	3 M H_2_SO_4_	1.4	1 A g^–1^	76.8	20.9	491.5	90.3	5000	^65^
Ti_3_C_2_T_*x*_/AC//Ti_3_C_2_T_*x*_/AC	PVA/H_2_SO_4_	1.2	0.5 A g^–1^	285.59	57.89	300	92.98	5000	^66^
M/PANI-5//CB	**1 M TEABF**_**4**_ **/DMSO**	**1.4**	**1**A g^**–1**^	**384.9**	**104.8**	**1400**	**85**	**10000**	**This work**

a MOF-metal organic framework; rGO-reduced graphene oxide; AC-activated carbon; V-PMOF- V_2_CT_*x*_ MXene based porphyrinic MOF; PANI-polyaniline; LDH-layered double hydroxides; CB-carbon black.

Three asymmetric supercapacitors connected in series were used to power a 3 V red LED, further demonstrating the device’s practical viability (Figure S9d) and Figure 7(d)). The successful illumination confirms the device’s ability to provide sufficient energy output and operating voltage for real-time applications, highlighting its potential for portable and low-power electronics.

The synergistic interaction of Mo_2_Ti_2_C_3_ MXene and PANI is the source of the M/PANI-5 composite’s outstanding electrochemical performance. PANI adds extra pseudocapacitance through reversible redox transitions, while Mo_2_Ti_2_C_3_ nanosheets mainly store charge through rapid EDLC resulting from reversible electrolyte ion adsorption and desorption throughout the charging and discharging procedure. Concurrently, the in situ polymerized PANI successfully inhibits the MXene layers’ restacking, revealing more electrochemically active spots and enhancing electrolyte accessibility. The two components’ close interfacial contact creates a continuous conductive network that shortens ion diffusion paths, reduces charge-transfer resistance, and speeds up electron transport. The M/PANI-5 electrode’s high specific capacitance, superior rate capacity, and stability over long-term cycling are the result of these synergistic effects, which also encourage quick electrochemical kinetics, effective use of active sites, and outstanding structural stability.

## 4 Conclusions

This work demonstrates the potential of DTM M/PANI polymer nanocomposites for supercapacitor applications. The successful synthesis of MXene and its subsequent integration with PANI through oxidative polymerization were confirmed through comprehensive structural and spectroscopic analyses. XRD patterns, together with SEM observations, confirm that the Al layer was successfully removed from the parent MAX phase and that layered MXene with a distinctive sheet-like shape was formed. The incorporation of PANI within the MXene framework is evidenced by the presence of characteristic PANI diffraction features in XRD and the quinoid and benzenoid vibrational bands in FTIR spectra. SEM and HRTEM analyses further reveal that PANI is uniformly distributed over the MXene nanosheets, forming a well-integrated composite structure, while EDS elemental mapping confirms the homogeneous distribution of Mo, Ti, C, N, O, and S elements. XPS analysis provides additional confirmation of composite formation, with C 1s and N 1s spectra indicating the presence of C–N linkages and characteristic presence of different nitrogen environments, including amine (−NH−) and imine (−N=) groups, characteristic of the PANI backbone, alongside the MXene elements. Particularly, the shift of the (002) peak toward lower angles for M/PANI-5 in XRD suggests an increase in interlayer spacing upon PANI incorporation, which effectively suppresses restacking of MXene sheets and facilitates improved ion transport. Among the studied compositions, the M/PANI-5 demonstrates optimal structural integration, balancing PANI content around MXene nanosheets. The asymmetric device fabricated keeping the optimum M/PANI-5 composite as positive electrode exhibited a specific capacitance of 384.9 F g^–1^ at 1 A g^–1^, along with an energy density of 104.8 Wh kg^–1^ and a power density of 1400 W kg^–1^, demonstrating practical applicability of the material. Overall, the well-interconnected MXene-PANI architecture enhances structural stability and provides efficient pathways for charge transport, making the composite a promising electrode material for high-performance supercapacitor applications.

## Supplementary Material



## Notes

Characterization techniques and electrochemical measurements; crystallite size and interlayer spacing of the prepared MXene and composites (Table S1); XRD spectra of freshly prepared and 9-month-old Mo_2_Ti_2_C_3_ MXene (Figure S1); Raman spectra of Mo_2_Ti_2_C_3_, PANI, and their composites (Figure S2); SEM images and EDS analysis of pristine PANI (Figure S3); composite samples with different ratios (Figures S4–S6); along with elemental mapping; XPS survey and high-resolution spectra of MXene are presented in Figure S7; electrochemical (log I–V) behavior is shown in Figure S8; Figure S9: shows digital photographs of the device fabrication and LED demonstration (PDF)
