# Supplementary material for: Double-Transition-Metal Mo2Ti2C3 Mxene-Polyaniline Composites for High-Performance Supercapacitors
Source: ACS Omega. 2026 Sep 10;11(37):56145–58. doi: 10.1021/acsomega.6c06468 (PMC13613443; doi:10.1021/acsomega.6c06468)
Supplement: Supplementary file 1 [file ao6c06468_si_001.pdf]

Supporting Information

Double-Transition Metal $Mo_2Ti_2C_3$ MXene-Polyaniline composites for High-Performance Supercapacitors

Nisha H. Makani[1#], Shrabani De[2#], Mia Thompson[1], Peshal Karki[1], Bishnu Bastakoti[2], and Bhoj Raj Gautam[1*]

[1]*Department of Chemistry, Physics and Materials Science, Fayetteville State University, Fayetteville, North Carolina 28301, United States of America*

[2]*Department of Chemistry, North Carolina A&T State University, Greensboro, NC 27411, United States of America*

Email: bgautam@uncfsu.edu

1. Characterization Techniques:

The crystal structure and phase purity of the samples were analyzed using X-ray diffraction (XRD) on a Rigaku MiniFlex diffractometer employing Cu Kα radiation (λ = 1.5406 Å) over an appropriate 2θ range (3°-60°) at room temperature. The surface morphology and microstructural features were examined using a field-emission scanning electron microscope (FE-SEM, JEOL) operated at an accelerating voltage of 20 kV under high vacuum. Elemental composition and mapping were determined using energy-dispersive X-ray spectroscopy (EDS) integrated with SEM. High-resolution transmission electron microscopy (HRTEM) on a JEOL 2100PLUS, operated at an accelerating voltage of 200 kV and combined with EDS, was utilized for detailed structural and compositional analysis. Fourier-transform infrared (FTIR) spectroscopy was carried out using a Horiba FTIR spectrometer in ATR mode. X-ray photoelectron spectroscopy (XPS) measurements were carried out using a Kratos Axis Supra spectrometer equipped with a monochromatic Al Kα radiation source (hν = 1486.6 eV) to investigate the surface chemical composition of the pristine MXene and M/PANI-5 sample.

2. Electrochemical measurements:

All electrochemical measurements, including both three-electrode and two-electrode configurations, were conducted using a BioLogic VSP-50 electrochemical workstation. The electrochemical performance in the three-electrode configuration was evaluated in 1 M $TEABF_4$ dissolved in DMSO as the electrolyte. Electroactive-material-coated carbon cloth served as the working electrode, while an Ag/AgCl electrode and a platinum (Pt) wire were used as reference and counter electrodes, respectively. The working electrode was prepared by drop-casting a homogeneous slurry consisting of the synthesized electroactive material (80 wt%), carbon black (10 wt%), and poly(vinylidene fluoride) (PVDF, 10 wt%) dispersed in N-methyl-2-pyrrolidone (NMP) onto a 1 cm × 1 cm carbon cloth substrate, followed by drying in vacuum at 60 °C. The mass loading of the active material was

approximately 0.3 mg. Cyclic voltammetry (CV) and galvanostatic charge-discharge (GCD) measurements were performed within a potential window of 0.0 V - 1.1 V to investigate the electrochemical behavior of the prepared electrodes. Electrochemical impedance spectroscopy (EIS) was carried out over a frequency range from 1 MHz to 1 Hz with an AC perturbation amplitude of 10 mV at room temperature. The specific capacitance (C, F g^{-1}) was calculated from the GCD curves according to Eq. (1)[1].

$$C = \frac{i \times \Delta t}{m \times \Delta V} \tag{1}$$

Where, *i* is the charging-discharging current, Δt is the time required in seconds for the discharge cycle, *m* is the mass of the active material, and ΔV is the voltage range.

The asymmetric supercapacitor (ASC) device was fabricated by coating a homogeneous slurry of the electroactive material (with the same composition as described above) onto a 3 cm × 3 cm carbon cloth substrate and evaluated in a two-electrode configuration. The assembled device employed the optimized M/PANI-5 composite as the positive electrode, while carbon black was used as the negative electrode. A Whatman 42 filter paper served as the separator. The electrochemical performance of the assembled ASC device was investigated within an operating voltage window of 0.0-1.4 V in 1 M $TEABF_4$ dissolved in DMSO electrolyte. The active mass loading of the positive electrode was approximately 4.5 mg. The specific capacitance (C in F g^{-1}) of the device was calculated using Eq. 2, where m is the mass of positive electrode. The specific energy density (E in W h kg^{-1}) and power density (P in W kg^{-1}) of the device were evaluated using Eq. 3, and Eq. 4[2], respectively:

$$C = \frac{2 \times i \times \Delta t}{m \times \Delta V} \tag{2}$$

$$E = \frac{C\Delta V^2}{7.2} \tag{3}$$

$$P = \frac{E \times 3600}{\Delta t} \tag{4}$$

The stored charge (Q) is dependent on specific capacitance (C), voltage window (ΔV), and mass of the active material (m) of the corresponding electrode[3]

$$Q = C \times \Delta V \times m \tag{5}$$

When the charge equivalence ($Q_+=Q_-$) is applied, the mass ratio (m_+/m_-) of the positive and negative electrode can be calculated using the following equation[3].

$$\frac{m_+}{m_-} = \frac{C_- \, \Delta V_-}{C_+ \, \Delta V_+} \tag{6}$$

The calculated mass ratio of positive and negative electrode using Eq. 6 is 0.23.

Table S1: Crystallite size and interlayer spacing of prepared MXene and composites.

Sample	Crystallite Size (nm)	Interlayer spacing (nm)
$Mo_2Ti_2C_3$	10.88	1.633
M/PANI-10	28.78	1.568
M/PANI-5	4.36	1.861
M/PANI-2.5	7.98	1.519

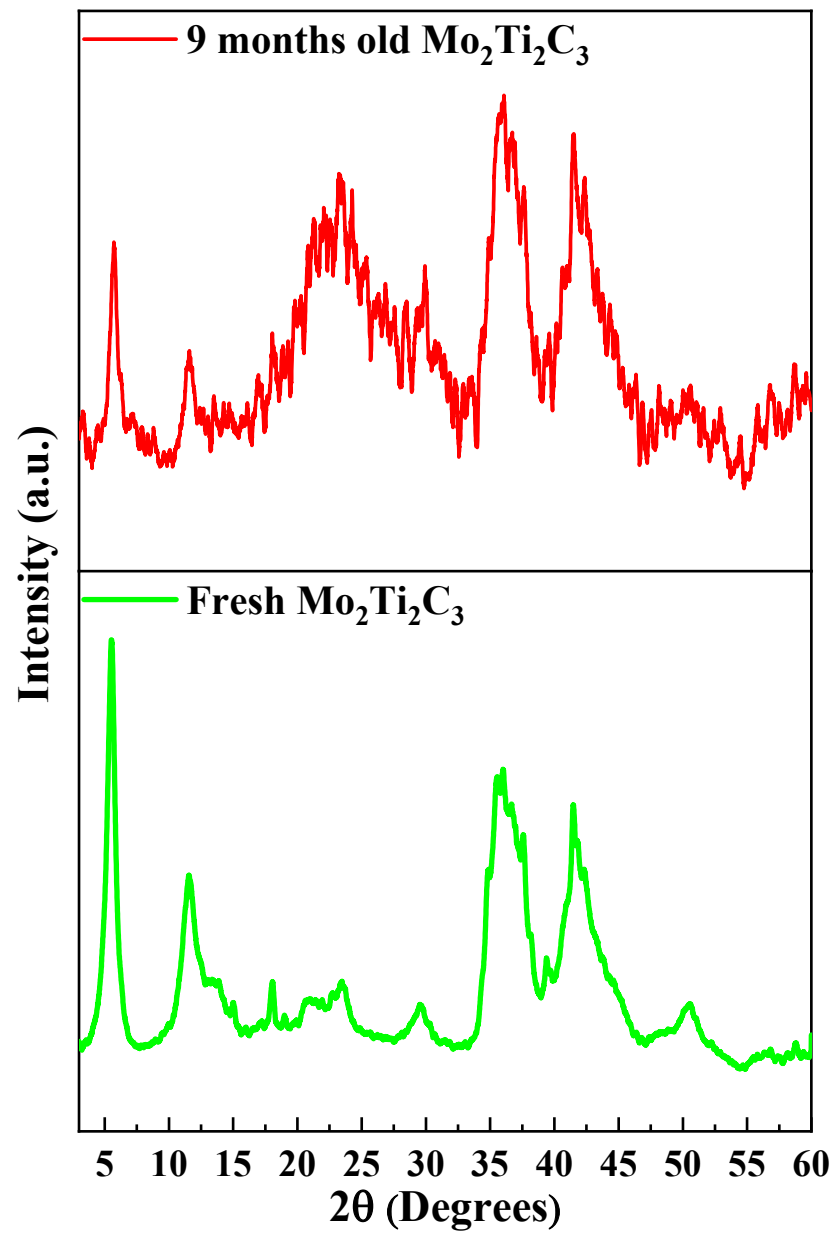

Figure S1: *XRD spectra of freshly prepared and 9-month-old $Mo_2Ti_2C_3$ MXene*

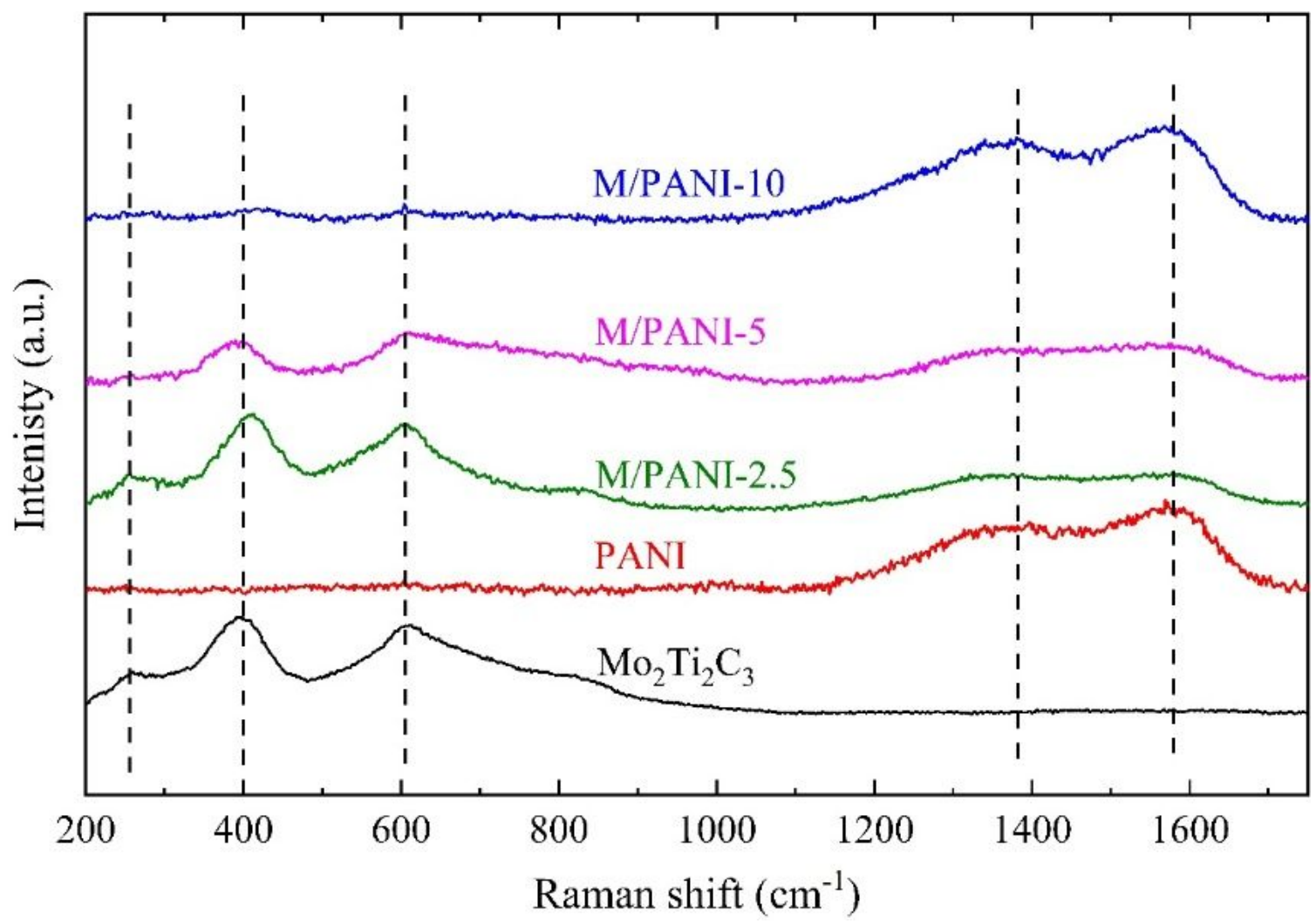

Figure S2: *Raman spectra of $Mo_2Ti_2C_3$, PANI, and their composites (M/PANI-2.5, M/PANI-5 and M/PANI-10)*

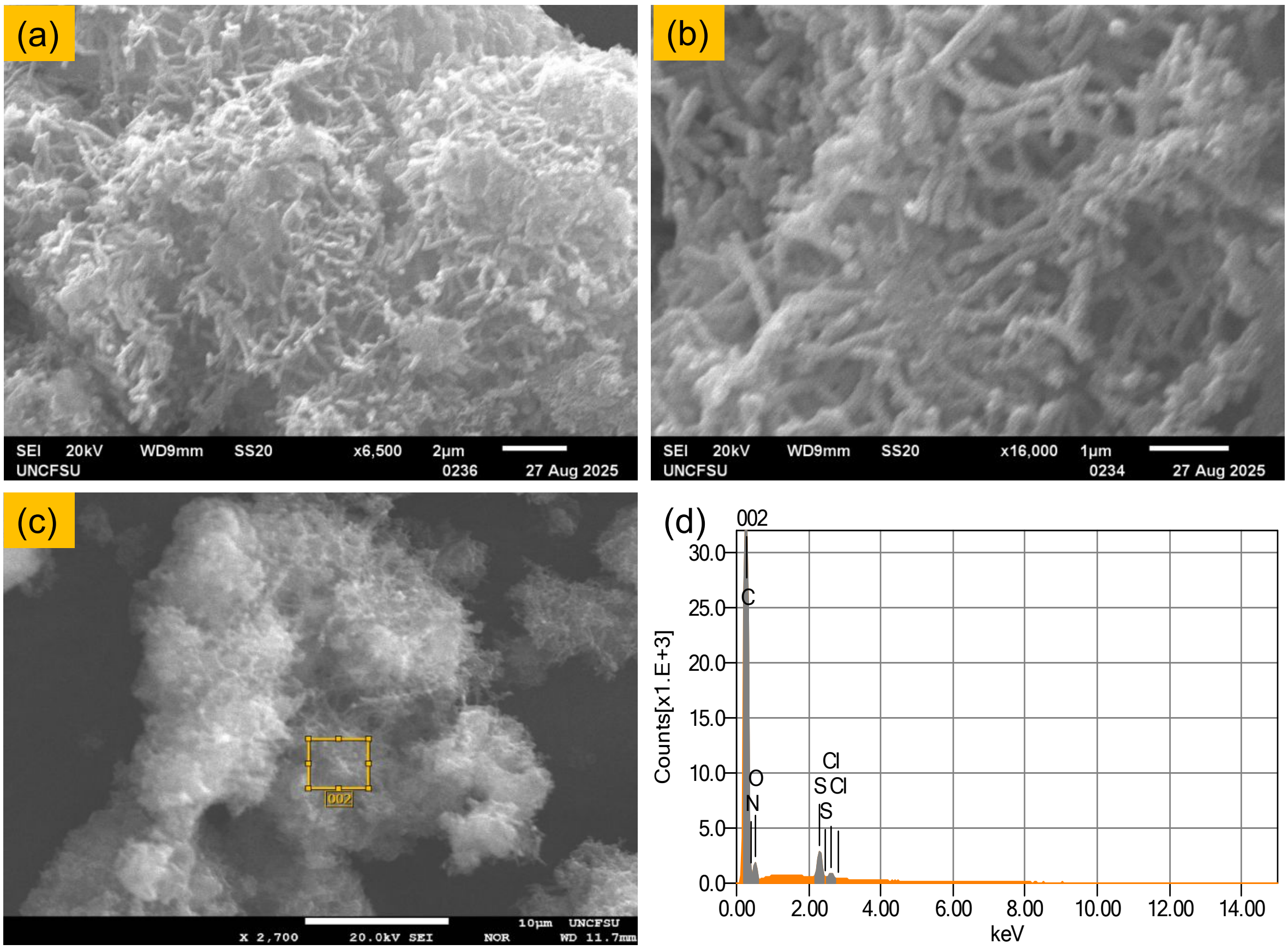

Figure S3: *SEM images of pristine PANI at (a) low magnification and (b) high magnification; (c) SEM image of PANI highlighting the region selected for EDS analysis, and (d) EDS spectrum corresponding to the selected area.*

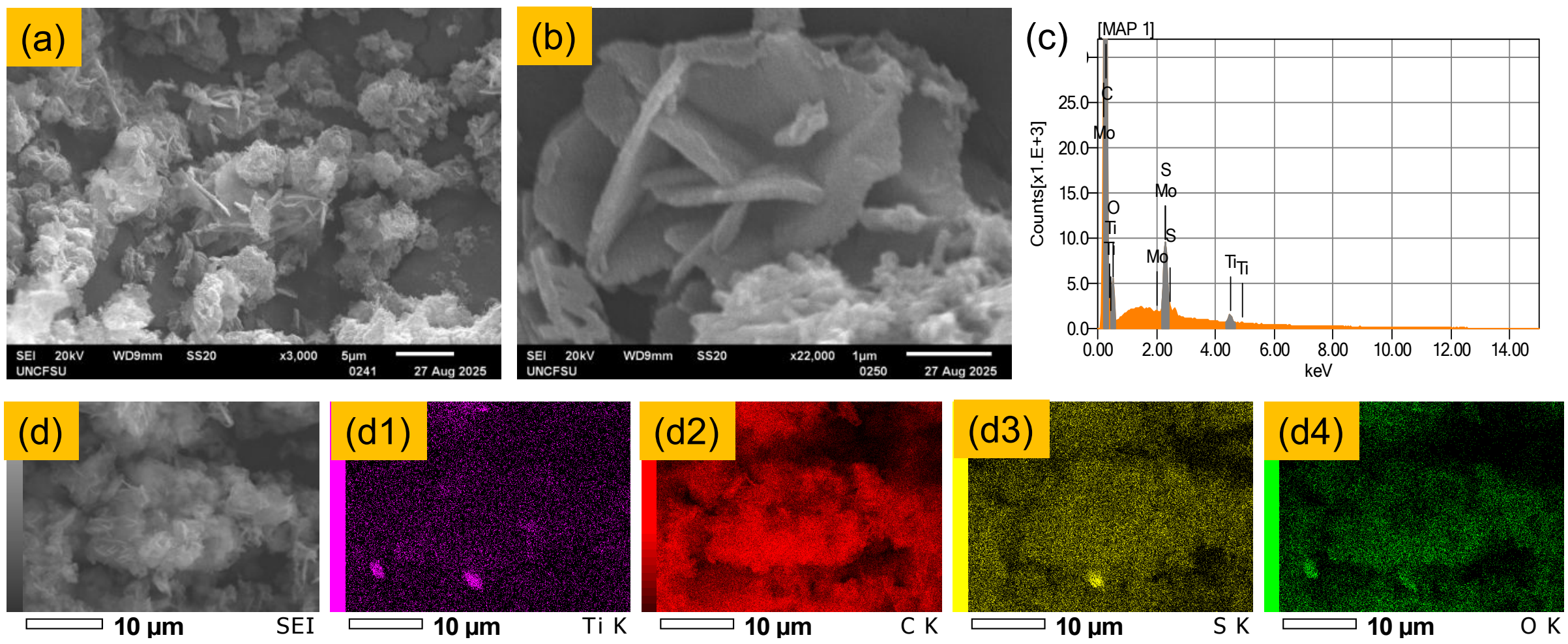

Figure S4: *SEM images of the synthesized M/PANI-10 composite showing (a) low and (b) high magnification, (c) EDS spectrum of the composite; (d) SEM image of the selected region for elemental mapping analysis, and (d1-d4) corresponding elemental distribution maps of Ti, C, S, and O, respectively.*

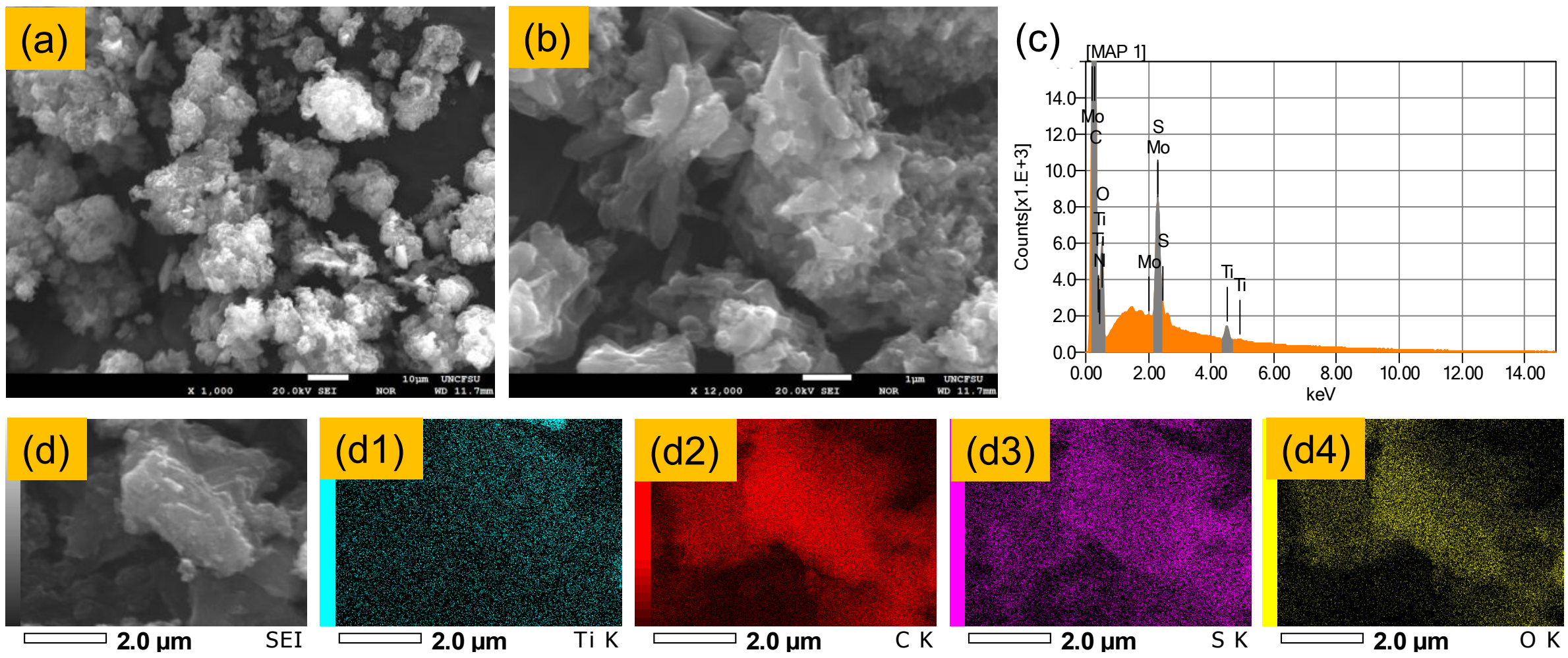

Figure S5: *SEM images of the synthesized M/PANI-5 composite showing (a) low and (b) high magnification, (c) EDS spectrum of the composite; (d) SEM image of the selected region for elemental mapping analysis, and (d1-d4) corresponding elemental distribution maps of Ti, C, S, and O, respectively.*

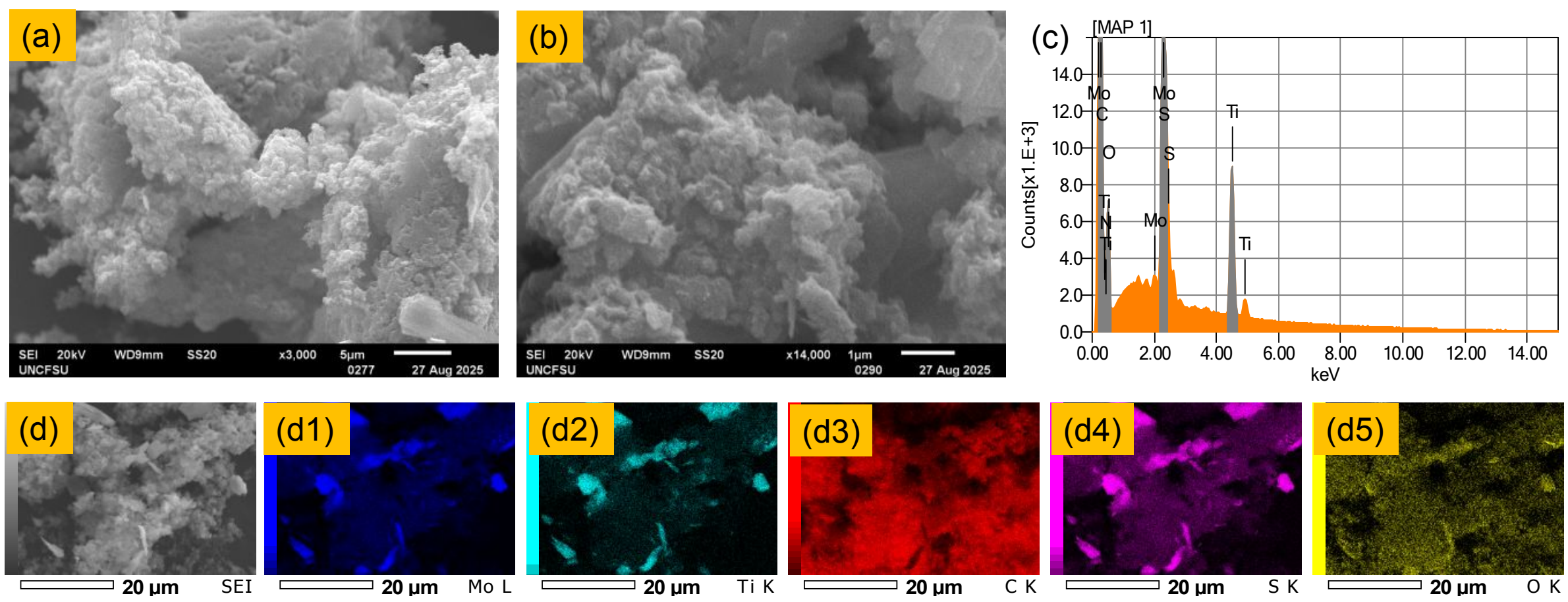

Figure S6: *SEM images of the synthesized M/PANI-2.5 composite showing (a) low and (b) high magnification, (c) EDS spectrum of the composite; (d) SEM image of the selected region for elemental mapping analysis, and (d1-d5) corresponding elemental distribution maps of Mo, Ti, C, S, and O, respectively.*

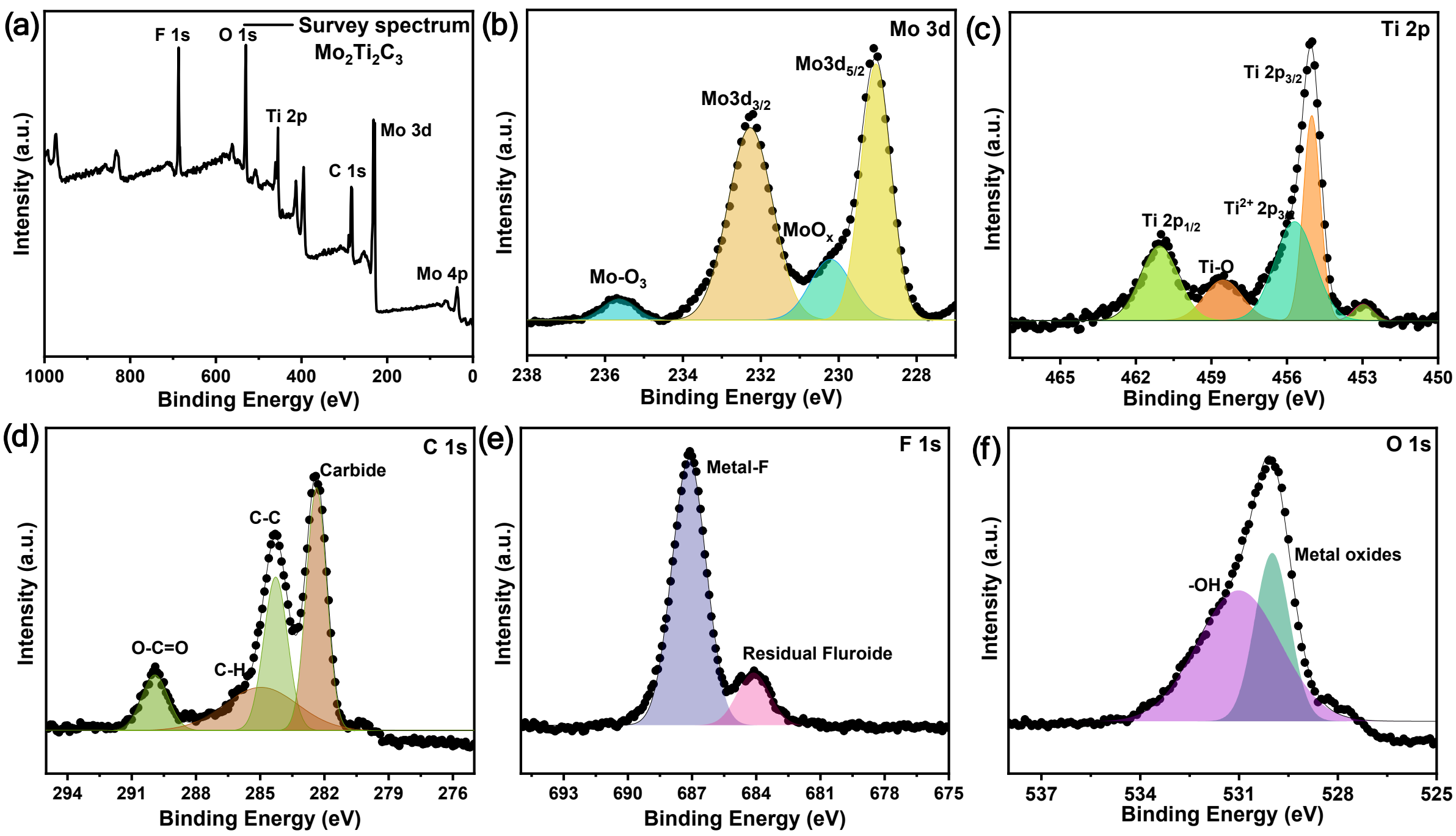

Figure S7: *(a) XPS survey spectrum of MXene and the corresponding high-resolution spectra of (b) Mo, (c) Ti, (d) C, (e) F, and (f) O, respectively.*

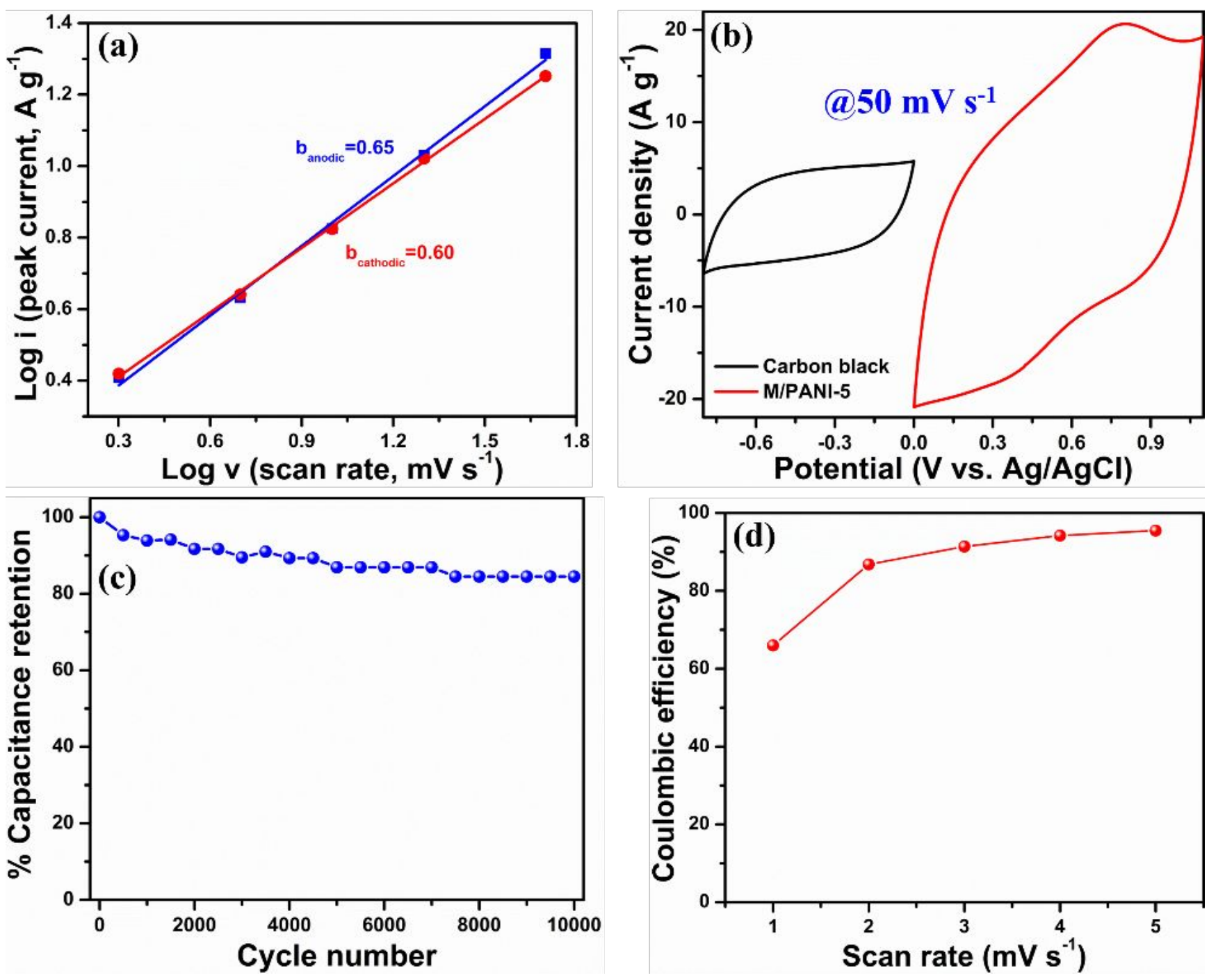

Figure S8: *(a) log (i) vs. log (v) profile of M/PANI-5, (b) CV profiles of carbon black at negative potential range and M/PANI-5 at positive potential range, (c) cyclic stability of the M/PANI-5 electrode in the three-electrode configuration, and (d) coulombic efficiency of the asymmetric supercapacitor at different scan rates.*

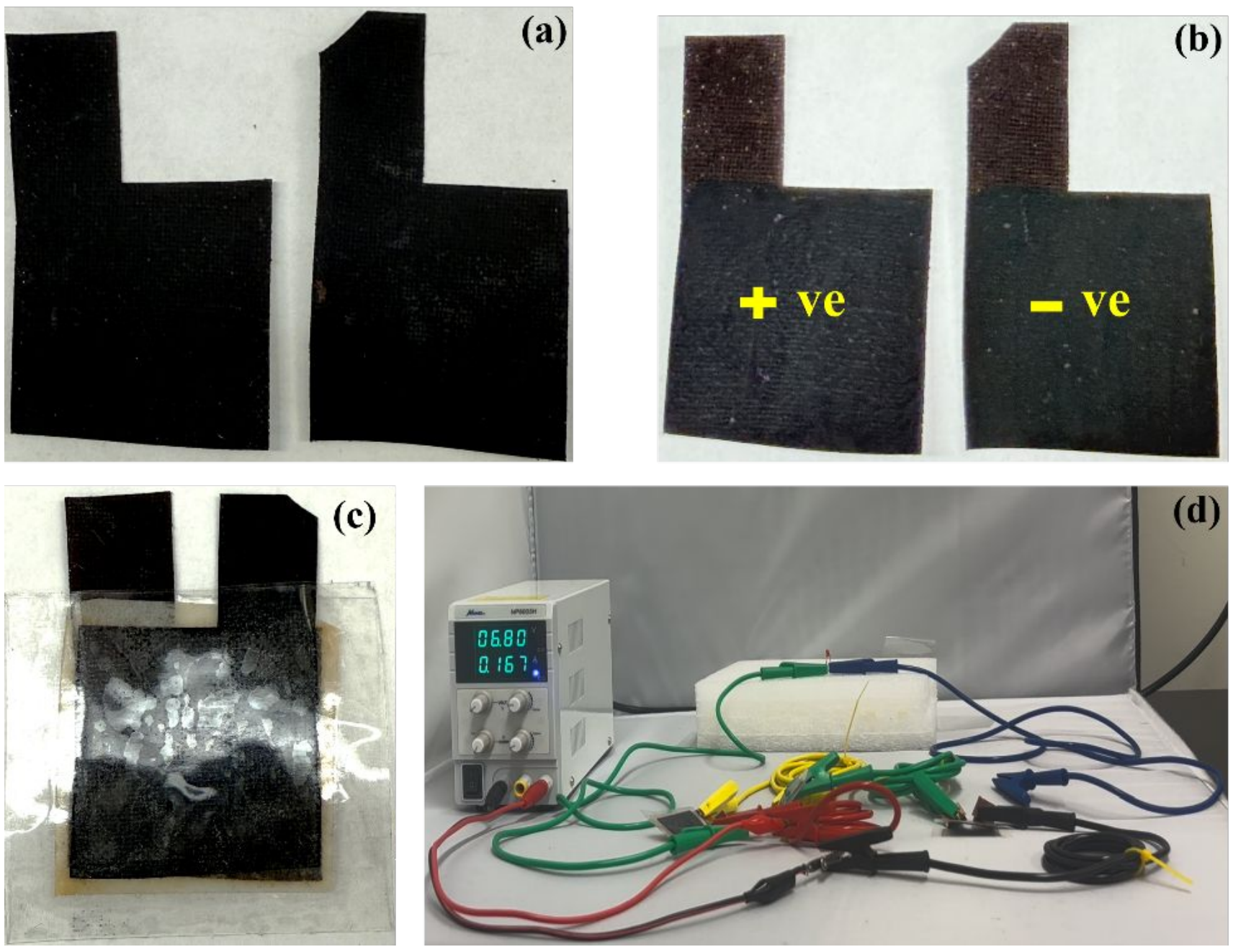

Figure S9. *Digital photographs of (a) bare carbon cloth (current collector), (b) electroactive material coated carbon cloth, (c) fabricated asymmetric supercapacitor device, and (d) charging setup of lighting up LED.*

References:

1. De, S.; Maity, C. K.; Sahoo, S.; Nayak, G. C. Polyindole Booster for Ti3C2TxMXene Based Symmetric and Asymmetric Supercapacitor Devices. *ACS Appl. Energy Mater.* 2021, *4* (4), 3712–3723.
2. De, S.; Roy, S.; Nayak, G. C. MoSn2Se4-Decorated MXene/Functionalized RGO Nanohybrid for Ultrastable Supercapacitor and Oxygen Evolution Catalyst. *Mater. Today Nano* 2023, *22*, 100337.
3. Redondo, E.; Fevre, L. W. L.; Fields, R.; Todd, R.; Forsyth, A. J.; Dryfe, R. A. W. Enhancing Supercapacitor Energy Density by Mass-Balancing of Graphene Composite Electrodes. *Electrochim. Acta* 2020, *360*.
